# Sleep staging in the ICU with heart rate variability and breathing signals. An exploratory cross-sectional study using deep neural networks

**DOI:** 10.3389/fnetp.2023.1120390

**Published:** 2023-02-27

**Authors:** Wolfgang Ganglberger, Parimala Velpula Krishnamurthy, Syed A. Quadri, Ryan A. Tesh, Abigail A. Bucklin, Noor Adra, Madalena Da Silva Cardoso, Michael J. Leone, Aashritha Hemmige, Subapriya Rajan, Ezhil Panneerselvam, Luis Paixao, Jasmine Higgins, Muhammad Abubakar Ayub, Yu-Ping Shao, Brian Coughlin, Haoqi Sun, Elissa M. Ye, Sydney S. Cash, B. Taylor Thompson, Oluwaseun Akeju, David Kuller, Robert J. Thomas, M. Brandon Westover

**Affiliations:** ^1^ Department of Neurology, Massachusetts General Hospital, MGH, Boston, MA, United States; ^2^ Clinical Data Animation Center (CDAC), Massachusetts General Hospital, Boston, MA, United States; ^3^ Sleep and Health Zurich, University of Zurich, Zurich, Switzerland; ^4^ Henry and Allison McCance Center for Brain Health, Massachusetts General Hospital, Boston, MA, United States; ^5^ Harvard Medical School, Boston, MA, United States; ^6^ Department of Medicine, Massachusetts General Hospital, Boston, MA, United States; ^7^ Department of Anesthesia, Critical Care and Pain Medicine, Massachusetts General Hospital, Boston, MA, United States; ^8^ MyAir Inc, Boston, MA, United States; ^9^ Beth Israel Deaconess Medical Center, Department of Medicine, Division of Pulmonary, Critical Care and Sleep, Boston, MA, United States

**Keywords:** sleep, sleep staging, intensive care unit (ICU), deep learning-artificial neural network, artificial intelligence-AI, heart rate variability (HRV), respiration

## Abstract

**Introduction:** To measure sleep in the intensive care unit (ICU), full polysomnography is impractical, while activity monitoring and subjective assessments are severely confounded. However, sleep is an intensely networked state, and reflected in numerous signals. Here, we explore the feasibility of estimating conventional sleep indices in the ICU with heart rate variability (HRV) and respiration signals using artificial intelligence methods

**Methods:** We used deep learning models to stage sleep with HRV (through electrocardiogram) and respiratory effort (through a wearable belt) signals in critically ill adult patients admitted to surgical and medical ICUs, and in age and sex-matched sleep laboratory patients

**Results:** We studied 102 adult patients in the ICU across multiple days and nights, and 220 patients in a clinical sleep laboratory. We found that sleep stages predicted by HRV- and breathing-based models showed agreement in 60% of the ICU data and in 81% of the sleep laboratory data. In the ICU, deep NREM (N2 + N3) proportion of total sleep duration was reduced (ICU 39%, sleep laboratory 57%, *p* < 0.01), REM proportion showed heavy-tailed distribution, and the number of wake transitions per hour of sleep (median 3.6) was comparable to sleep laboratory patients with sleep-disordered breathing (median 3.9). Sleep in the ICU was also fragmented, with 38% of sleep occurring during daytime hours. Finally, patients in the ICU showed faster and less variable breathing patterns compared to sleep laboratory patients

**Conclusion:** The cardiovascular and respiratory networks encode sleep state information, which, together with artificial intelligence methods, can be utilized to measure sleep state in the ICU

## 1 Introduction

The human body is composed of multiple physiological and organ systems, each with its own structure and function (Bashan et al., 2012; Ivanov and Bartsch, 2014; Ivanov, 2021). In a complex multi-component networked state, healthy sleep is a biological imperative (Mukherjee et al., 2015; Kryger et al., 2017). Breakdown of this sleep network impairs critical brain and body functions, including memory (Rasch and Born, 2013), learning, attention, and affective state (Banks and Dinges, 2007; Killgore, 2010; Mueller et al., 2013; Naiman, 2017; Holding et al., 2019), and regulation of blood pressure (Khan and Aouad, 2017), inflammatory processes (Besedovsky et al., 2019; Irwin, 2019), metabolic control (Leproult and Cauter, 2010; Philip et al., 2012; Ma et al., 2015), and stress responses (Afolalu et al., 2018; Dunietz et al., 2018; Krause et al., 2019). The intensive care unit (ICU) is associated with disrupted sleep, due to internal (e.g., pain, immunocompromisation, dyspnea, and apnea) and external (e.g., noise, circadian mismatch) factors. The network of sleep can be so distorted in the ICU that conventional sleep stages can be hard to recognize (Watson et al., 2013). Sleep disruption in the ICU contributes to delirium (Watson et al., 2012), difficult weaning from mechanical ventilation (Hardin, 2009; Thille et al., 2018; Dres et al., 2019), and increased risk of autonomic, inflammatory, and metabolic dysfunction (Weinhouse and Schwab, 2006).

Despite the urgency of improving sleep in the ICU, measuring sleep in this environment is challenging since conventional polysomnography is difficult to operationalize in the ICU setting (Boyko et al., 2017). Subjective sleep estimates and movement analysis using actigraphy can provide crude assessments of sleep (Boyko et al., 2017; Schwab et al., 2018), but are heavily confounded by common ICU experiences, including sedation, monitoring, illness, and immobility. No present method of measuring sleep in the ICU is satisfactory (Darbyshire et al., 2020), thus alternative approaches are needed.

In an effectively networked system, key information content can often be derived from subcomponents, without having access to all of the system. Some standard sleep state signals are readily acquired in most medical circumstances such as the electrocardiogram and respiration. Our first aim for this study was to evaluate the validity of monitoring sleep in ICU patients using easily obtainable biosignals, such as electrocardiogram (ECG) and respiration, using artificial intelligence methods. Although sleep states are commonly discerned through electroencephalogram (EEG) signals, they can also be decoded through analysis of non-EEG signals (Radha et al., 2019; Sun et al., 2019; Sridhar et al., 2020) since sleep modifies a variety of biosignals (Somers et al., 1993; Fink et al., 2018), including blood pressure, heart rate, and respiration. Additionally, compared to EEG, respiration and ECG measurements are easier to acquire and offer a more practical and repeatable diagnostic tool. Respiration and ECG signals likely also measure sleep more objectively compared to actigraphy and subjective assessments.

For this study, we used deep neural network models to estimate sleep stages by analysis of the networked interactions of cardiac, autonomic and respiratory systems, from heart rate variability (HRV), derived from ECG, and breathing signals, obtained with a single respiratory effort belt. To investigate the performance and behavior of the models, we analyzed how well the HRV and breathing models agreed in determining sleep stages both in ICU patients and in age and sex-matched patients referred to a clinical sleep laboratory. We further used the sleep laboratory dataset to compare sleep staging performances, both when the HRV- and breathing-based models agreed and disagreed, to the gold standard sleep stage annotations, which involves manual scoring of polysomnography EEG signals by experts. We also hypothesized that the HRV and breathing models would show larger disagreement compared to the sleep laboratory dataset given the extent of respiratory and cardiac physiology in critical illness. We evaluated whether specific HRV- and respiratory features, and variables such as medical conditions, severity of illness, and pharmacological drugs are associated with disagreement of these two sleep staging models. This is the first study investigating sleep stages in the ICU with non-EEG biosignals. While future studies need to follow up with additional analysis on this line of research, including EEG analysis in ICU patients, in the present study we estimate to what extent electrocardiogram and respiration signals, together with machine learning methods, may be able to assist with sleep analysis in clinics already today.

The second aim of this study was to determine common sleep statistics and respiratory variables in ICU patients, as well as in non-critically ill patients undergoing overnight diagnostic polysomnography recordings for suspected sleep disorders in the sleep laboratory. We tested robustness of our results through sensitivity analysis.

Finally, fragmentation and loss of cohesion of the sleep network is inevitable in the ICU (Freedman et al., 2001), which prevents patients from getting adequate consolidated periods of rest. As such, the last aim of this study was to quantify sleep fragmentation in patients admitted to the ICU.

## 2 Materials and methods

### 2.1 Study oversight

Patients were enrolled after written consent in a randomized clinical trial, Investigation of Sleep in the Intensive Care Unit (NCT03355053 (https://clinicaltrials.gov/ct2/show/NCT03355053?term=Investigation+of+Sleep+in+the+Intensive+Care+Unit, n.d.)), at the Massachusetts General Hospital (MGH) from June 2018 to November 2019. The clinical trial involved randomizing patients into three groups, where two of the groups received a low dose of dexmedetomidine (0.1 or 0.3 mcg/kg/h) overnight continuously for 11 h, and the third group received placebo (normal saline). Exclusion criteria for the clinical trial include severe dementia, known pre-existing neurologic diseases or cognitive deficits, serious cardiac disease, severe liver dysfunction, severe renal dysfunction, and low likelihood of survival for 24 h-all criteria can be found in the online supplement. The study was approved by the Mass General Brigham Institutional Review Board and in accordance with the Declaration of Helsinki.

### 2.2 Dataset-ICU Cohort

The sample size for this study was determined by starting with enrolled patients and then excluding all patients under 45 years of age and patients who had less than 2 h of ECG or respiratory data. Patients were non-mechanically ventilated at the time of enrollment, although some were subsequently mechanically ventilated during the course of hospitalization; see Table 1. At the start of the trial, a respiratory belt (*Airgo*, a CE Class IIa certified wearable medical device (MyAir LLC, n.d.), Supplementary Figure S1) was placed around a patient’s chest, as close to the floating ribs as possible, until they were transferred outside of the ICU. The belt contains a conductive silver band that measures respiratory effort by sensing changes in electrical resistance that correspond to changes in belt length induced by thoracic movements. The amplitude values of the belt were not calibrated. The sampling frequency was 10 Hz. Patients did not wear the belt when mechanically ventilated. In addition to demographic data, we collected information regarding labs, medications, vital signs, and ICD-10 codes from the hospital’s electronic medical records. Vital signs at higher time resolution (0.5 Hz) and electrocardiogram (ECG) (256 Hz) data were collected from the bedside telemetry monitors over the hospital network using BedMaster software (Excel Medical, Jupiter, FL). Signals collected through bedside monitors and the wearable respiratory device both contained real-time timestamps; correct alignment was manually reviewed for all patients. Charlson Comorbidity Index (Charlson et al., 1994) and Sequential Organ Failure Assessment (SOFA) (Vincent et al., 1996) scores were computed.

**TABLE 1:** Baseline characteristics.

	ICU n (%)	Sleep laboratory n (%)	*p*-value
Number of Patients	102	220	0.80
Age (years)			
Mean (Std)	68 (9)	68 (9)	
Range	50–88	51–101	
Sex			0.93
Male	61 (60)	122 (55)	
Female	41 (40)	98 (45)	
Race			0.99
White or Caucasian	92 (90)	152 (69)	
Black or African American	5 (5)	9 (4)	
Asian	2 (2)	7 (3)	
American Indian or Alaska Native	1 (1)	1 (0.5)	
Unknown	2 (2)	51 (23)	
Ethnicity			0.92
Non-Hispanic	95 (93)	168 (76)	
Hispanic	2 (2)	6 (3)	
Unknown	5 (5)	46 (21)	
BMI (kg/m^2^)^a^	27 (6)	31 (6)	1e-6
Wearable Belt Length (cm)^a^	89 (12)	n/a	
Charlson Comorbidity Index	2.2 (2.1)	1.9 (1.7)	0.11
Apnea-Hypopnea-Index	n/a	9 (9)	
Previous OSA Diagnosis	28 (27)	n/a	
History of CHF	29 (28)	n/a	
History of COPD	31 (30)	n/a	
ICU Type			
Medical	33 (34)	n/a	
Surgical	68 (66)	n/a	
SOFA Score at first study day			
Mean (Std)	3.1 (2.6)	n/a	
Range	0–11	n/a	
Primary and/or secondary diagnosis			
Acute Kidney Injury	33 (32)	n/a	
Shock	30 (29)	n/a	
Respiratory Failure	21 (21)	n/a	
Anemia	20 (20)	n/a	
Sepsis	19 (19)	n/a	
Pneumonia	15 (15)	n/a	
Encephalopathy, Altered			
Mental Status	14 (14)	n/a	
Pneumothorax, Hemothorax			
Pulmonary Edema, Pleural			
Effusion	13 (13)	n/a	
GI perforation, incarcerated			
hernia, SBO, ischemic colitis	11 (11)	n/a	
Heart Failure	10 (10)	n/a	
Hemorrhage	10 (10)	n/a	
COPD, Interstitial lung disease	7 (7)	n/a	
Fall, Trauma, Burns	7 (7)	n/a	
Cirrhosis s/p liver transplant	6 (6)	n/a	
Other medical	34 (33)	n/a	
Other surgical	47 (46)	n/a	
In-hospital Mortality	0 (0)	n/a	
Three Month Mortality	19 (18)	n/a	
Readmission			
Hospital within 30 days	10 (10)	n/a	
ICU within 30 days	7 (7)	n/a	
Emergency department within 30 days	4 (4)	n/a	
Mechanical Ventilation			
During hospitalization	26 (25)	n/a	
Before enrollment	21 (20)	n/a	
During study period	7 (7)	n/a	
After 14 day study period	6 (6)	n/a	
Duration (days)^b^	0.5 (2.2)	n/a	
Medications usage within study period			
Opioids used	68 (68)	n/a	
Fentanyl equivalent (mg)^a^	38 (45)	n/a	
Benzodiazepines used	23 (23)	n/a	
Midazolam equivalent (mg)^a^	4 (6)	n/a	
Antipsychotics used	24 (24)	n/a	
DDD-method equivalent^a^	0.3 (0.4)	n/a	

^a^ Mean (Standard Deviation).

^b^ Median (Interquartile Range).

### 2.3 Dataset-Sleep laboratory cohort

404 patients who underwent overnight polysomnography (PSG) in the Massachusetts General Hospital sleep laboratory between January 2019 and January 2020 wore the same respiratory belt that was used in the ICU cohort. Participants were enrolled through verbal consent shortly before onset of the PSG. There were no exclusion criteria and enrollment stopped after reaching a target sample size of 404 patients. This study was also approved by the Mass General Brigham Institutional Review Board. To use sleep laboratory patients as control subjects for comparison with ICU patients, we applied the same exclusion criteria, i.e., excluding all patients under 45 years of age. We also balanced the distribution of age and sex between the ICU and sleep lab cohorts by applying k-nearest-neighbor matching, as previously described (Stuart, 2010). We further stratified sleep lab patients according to their Apnea-Hypopnea-Index (AHI) severity into no-disordered breathing (AHI <5) and disordered breathing (AHI >15) groups and applied the same matching method for each of these subgroups. Seven trained sleep technicians annotated PSG studies as part of routine clinical care according to American Academy of Sleep Medicine guidelines (Berry et al., 2012).

### 2.4 Biosignals preprocessing

Non-physiological data and data with low signal quality were removed from both the ECG and respiratory effort belt signal. For the respiratory signal, this was done with an algorithm checking for high and constant amplitude (belt not worn) and for absence of reliable breath detection (low signal quality). For each patient, we normalized the respiratory signal by subtracting the mean and dividing by the standard deviation calculated from the 1%–99% quantile clipped signal. We used the open-source PhysioNet Cardiovascular Signal Toolbox (Vest et al., 2019) to filter the ECG signal, extract R peaks based on the Pan Tompkins algorithm (Pan and Tompkins, 1985), and obtain a signal quality measure. We provide parameter settings for the preprocessing steps in the online supplement.

### 2.5 Sleep Staging

We used deep neural network models that use heart rate variability (a binary sequence with 1 for a detected R-peak in the ECG, 0 else) and respiratory effort signals as inputs to assign a sleep stage (Wake, R, N1, N2, and N3) to every 30-s epoch. We previously validated this approach on datasets from the MGH sleep laboratory and the Sleep Heart Health Study (Sun et al., 2019). Here, we used a model trained with signals from the wearable respiratory belt as input. For visual representation of the resulting sleep stages for all patients, swimmer plots were created.

### 2.6 Breathing features

From the respiratory belt’s signal, we computed four features using a moving window approach.

i. Respiratory rate (RR): number of breaths (inspiratory peaks) detected in 10 s (moving window) x 6.
ii. Inter-breath-interval (IBI): time (seconds) between two consecutive breaths.
iii. Ventilation coefficient of variation (ventilation CVar): we first computed a proxy of minute ventilation as the sum of positive amplitude changes (inspiration) over 10 s and scaled it to a reference of 1 minute (multiplied by 6), then computed the coefficient of variation over a 30-s window.
iv. Variability index: we computed the coefficient of variation of the IBIs over a 30-s window, and defined the variability index as: variability index = (ventilation CVar + IBI CVar)/2, i.e., the mean of the coefficient of variation computed from the breathing timing (IBI) and breathing amplitude (ventilation).

### 2.7 Statistical analysis-Sleep Staging

#### 2.7.1 Definition-concordant and discordant sleep

For all analyses in this study, we only used data where both HRV and breathing data was simultaneously available. We applied both the HRV and breathing-based sleep staging models individually on all data. Each 30 s epoch in the data was assigned a sleep stage by the HRV- and breathing-based models, yielding two hypnograms. Because disagreement in sleep staging in human experts is common (Danker-Hopfe et al., 2009), and because stages between wake, N1, N2, and N3 form a continuum, we defined *concordance* regarding the stage assigned to a given 30-s epoch of sleep if the models agreed to within one stage, and *discordance* if they did not. Specifically, we defined an ordinal progression of sleep depth for NREM sleep: W < N1 < N2 < N3, such that, e.g., if the HRV and breathing-based models assigned W and N1, respectively, this would be considered concordant; whereas assignments of W and N2 would be considered discordant. For REM sleep R, models were considered concordant only if both assigned a stage of R. For completeness, the full set of concordant and discordant stage assignments were.

*Concordance*: (W, W), (N1, N1), (N2, N2), (N3, N3), (R, R), (W, N1), (N1, N2), (N2, N3).
*Discordance*: (W, N2), (W, N3), (W, R), (N1, N3), (R, N1), (R, N2), (R, N3).

For the sleep lab polysomnography data, sleep staging agreements between the models and the experts were measured with confusion matrices and Cohen’s kappa (Cohen, 1960) for both concordant and discordant data.

#### 2.7.2 Sleep indices

We split each patient’s data into 24 h segments (*full day*), starting and ending at 08:00, and further defined *day* as 08:00–20:00 and *night* as 20:00–08:00. For every 24 h segment, we obtained the HRV-based and breathing-based hypnograms, and computed the following *sleep indices*.

1. Total sleep duration (in hours).
2. Concordant sleep duration (in hours).
3. Discordant sleep duration (in hours).
4. Proportion of discordant sleep from total sleep (in %).
5. Sleep fraction (%): sleep duration divided by amount of data available.
6. Stage R (%), Stage N1 (%), Stage N2 (%), Stage N3 (%), Stage N2 + N3 (%): Time spent in a specified sleep stage divided by sleep duration.
7. Sleep fragmentation index (SFI): Number of sleep stage transitions from (N2, N3, R) to (N1, W) divided by sleep duration.
8. Wake transitions/hour: Number of sleep stage transitions from (N1, N2, N3, R) to (W) divided by sleep duration.

We further computed mean sleep indices across the models:

$$x_{mean}=\left(x_{HRV}+x_{Breathing}\right)\;/\;2$$

where x is any of the 8 sleep indices listed above.

To ensure robust conclusions regarding sleep indices, we performed sensitivity analysis, by computing sleep indices using three complementary approaches. The approaches vary by what segments are included for sleep index computation, and by which part of sleep (total sleep or concordant sleep) is used to compute the sleep indices.

A1.  Inclusion criteria: Any amount of sleep. Sleep indices computed on total sleep.
A2. Inclusion criteria: At least 2 hours of concordant sleep. Sleep indices computed on total sleep.
A3. Inclusion criteria: At least 2 hours of concordant sleep. Sleep indices computed on concordant sleep.

For each approach, we carried out the following test procedure: For every subject we computed the mean sleep indices (mean x_mean_ across all 24 h segments). We then applied a Mann-Whitney U (MWU) test (H0: “equal distribution for both groups”) and Mood’s median test (H0: “equal medians for both groups”) to assess statistical difference between the ICU and sleep lab (overall and AHI subgroups) subjects. We considered sleep indices to be significantly different for two groups (Mann-Whitney U) with a significantly different effect direction (Mood’s median test) if both tests resulted in a *p*-value of less than 0.05. We chose to apply non-parametric tests instead of t-tests because we found that none of the data were normally distributed in either the ICU or sleep lab group, where we define non-normally distributed to mean that either the Shapiro-Wilk or D’Agostino’s K-squared test rejects null hypothesis of normality for alpha = 0.05. We report test results for all applied statistical tests.

#### 2.7.3 Sleep fragmentation in the ICU

We characterized sleep fragmentation in the ICU (Freedman et al., 2001) by the following metrics using HRV model-based sleep stages.

1. Proportion of day (08:00–20:00) spent asleep.
2. Proportion of night (20:00–08:00) spent asleep.
3. Proportion of sleep occurring in the day *versus* in the night.
4. Proportion of REM sleep occurring in the day *versus* in the night.
5. Number of sleep periods, with a duration of at least 1 minute, per 24 h.
6. Number of sleep periods, with a duration of at least 5 minutes, per 24 h.

#### 2.7.4 Subgroup analysis

The ICU patients were manually grouped according to their primary and main conditions, and the median sleep indices (HRV-based model, analysis approach A2) were computed for each group. The Kruskal-Wallis H test was applied to assess differences of individual sleep indices between groups.

#### 2.7.5 Latent feature representation of sleep

To assess similarities and differences in the latent feature representation between sleep epochs in the ICU and the sleep lab, we computed unsupervised UMAPs (Uniform Manifold Approximation and Projection) (McInnes et al., 2020) based on the sleep staging neural networks’ last hidden layer activations. UMAPs were created separately for the HRV-based model and breathing-based model across pooled sleep lab and ICU data, see supplement for details.

#### 2.7.6 Disagreement between HRV and breathing models-error analysis

We hypothesized that the following variables affect discordance in the HRV- and breathing-based models: 1) specific features from cardiac and respiratory signals (see supplement and Results); 2) daily dosing of opioids, benzodiazepines, and antipsychotics; 3) SOFA score, a measure of a patient’s illness severity. HRV and breathing features of interest were: RR interval, RR root mean square of successive differences (RMSSD), HRV very low frequency power (VLF), HRV low frequency power (LF), HRV high frequency power (HF), inter-breath-intervals, respiratory rate, respiratory variability index, ventilation CVar, cardiopulmonary coupling (Thomas et al., 2005) (CPC) low frequency coupling (LFC), CPC high frequency coupling (HFC). We computed these features for concordant and discordant sleep parts for each 24 h segment and performed a Mann-Whitney U test with a significance level of 0.01 for each feature pair. Next, we computed features for each 24 h segment and performed multilinear regression analysis with a LASSO penalty, with the features as independent variables and the discordant proportion (log-transformed) as the dependent variable. To test if daily administered doses of opioids (in Fentanyl milligram (Patanwala et al., 2007)), benzodiazepines (in Midazolam milligram (MacLaren and Sullivan, 2005; Guina and Merrill, 2018)), antipsychotics (DDD method (Leucht et al., 2016)) and the SOFA score were associated with discordance, we computed Pearson and Spearman correlations between each of these variables and the proportion of discordant sleep for each 24 h segment.

### 2.8 Statistical analysis-Breathing

For every 24 h segment with at least 2 hours of concordant sleep, we computed the breathing features as described above from concordant sleep and for each sleep stage. For each patient we averaged the features across all nights. For ICU and sleep lab cohorts, we computed the mean and standard deviation of each feature and assessed statistically significant differences between cohorts with t-tests for Gaussian features or Mann-Whitney U tests and Mood’s median test (analogous to sleep stage analysis) for non-Gaussian features.

Results are reported in accordance with the Strengthening the Reporting of Observational Studies in Epidemiology (STROBE) guidelines (Elm et al., 2007).

## 3 Results

### 3.1 Dataset

129 patients were enrolled in a clinical trial in the ICU at the Massachusetts General Hospital; we excluded two patients under 45 years old, and 25 patients for having less than 2 h of ECG or respiratory data available from our analysis, resulting in a cohort size of 102 patients (41 females, 61 males). In a sleep lab cohort, out of 404 enrolled patients, 97 were excluded due to age under 45 years, resulting in a sample size of 307 before matching. Matching resulted in 220 (98 females, 122 males) sleep lab patients, 77 (40 females, 37 males) with an AHI <5 and 52 (18 females, 34 males) with an AHI >15. Age distributions were similar for ICU and matched groups, both for male and female subjects; see Supplementary Table S1 for details. Table 1 summarizes the baseline characteristics of the ICU and the matched sleep lab cohort.

### 3.2 Biosignals preprocessing

After signal preprocessing, we obtained 6,728 h (280 days) of ECG data, 3,886 h (162 days) of breathing data, and 3,502 h (146 days) of simultaneous ECG and breathing data for the ICU cohort. For the sleep lab cohort, the numbers were 1,634, 1,609, and 1,562 h, respectively. Mean (SD) hours of data available per patient in the ICU (*N* = 103) were ECG 66.0 (27.8), Breathing 38.1 (28.1), for simultaneous ECG and breathing signals 34.3 (24.6), and for the sleep lab (*N* = 220) 7.4 (0.8), 7.3 (1.0), 7.1 (0.9) hours respectively.

### 3.3 Statistical analysis, Sleep Staging

**FIGURE 1:**
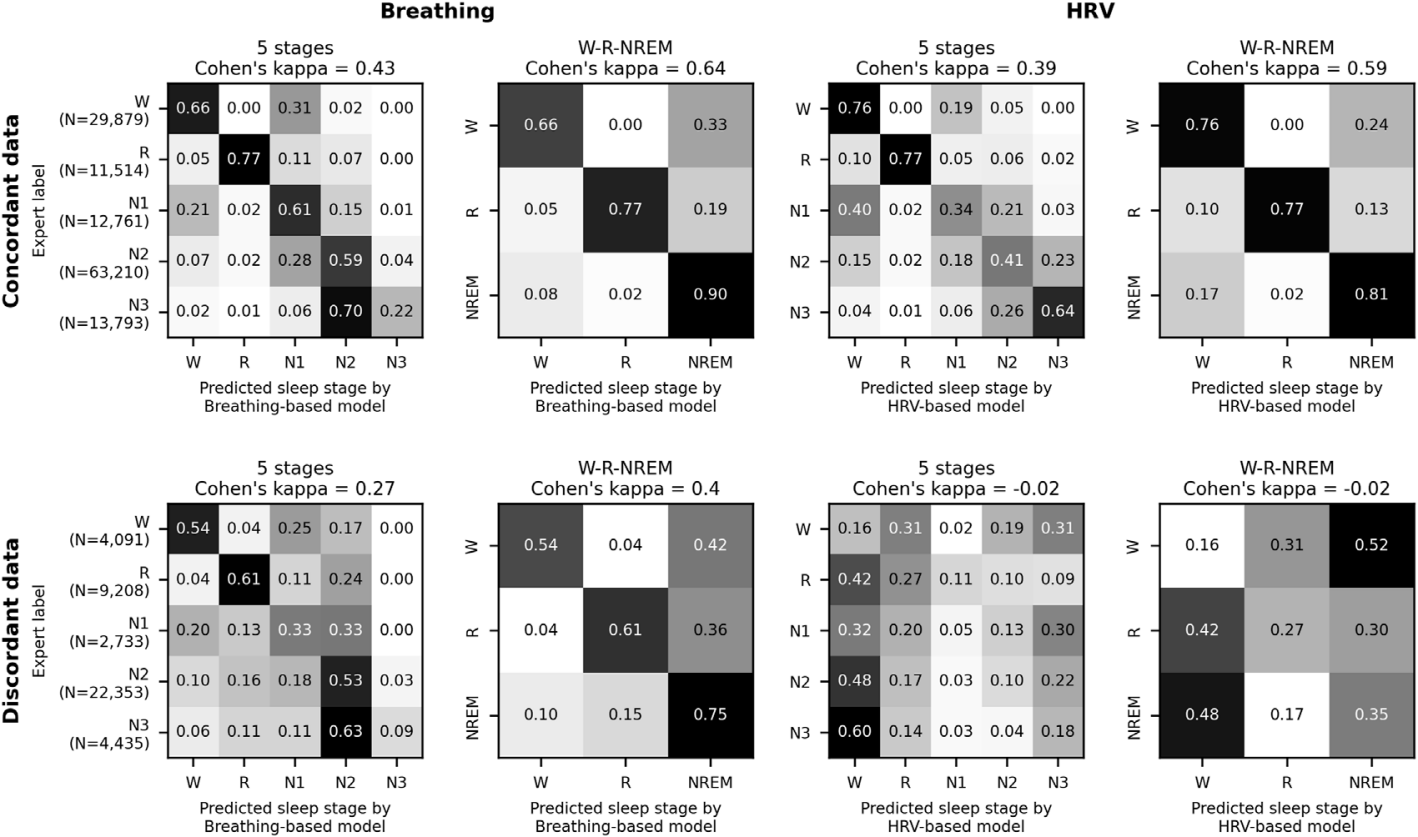
HRV-and breathing-based sleep stage concordance and human agreement. Model performance evaluation on 220 age and sex-matched sleep laboratory patients. Data where the HRV- and breathing-based sleep stages were in concordance (see main text for definition) also showed higher agreement with human expert labels. In total, 75.4% of all 30 s epochs were assigned concordant sleep stages by the models. For the discordant sleep epochs, the breathing-based sleep stages had markedly higher agreement with the expert labels than the HRV-based sleep stages.

In the sleep laboratory data, 131,157 out of 173,977 30-s epochs (75.4%) were assigned concordant sleep stages (see Methods) by the HRV- and breathing-based models. The models showed higher staging agreements with experts for concordant data than for discordant data, both when agreement was evaluated with Cohen’s kappa with AASM standard stages (W, R, N1, N2, N3), as well as with combined NREM stages (W, R, NREM), see Figure 1.

Sensitivity analysis, consisting of three analysis approaches resulted in the following total sleep times (TST), concordant sleep times (CST), and proportions sleep (S (%)) per 24 h segment (numbers given as mean (SD)):

A1. Inclusion: any sleep; sleep indices computed on total sleep.

ICU: 102 subjects (274 24-h segments), TST **6.2 (3.1)** hours, S (%) **50.4 (19.7)**
Sleep lab: 220 subjects (220 segments), TST **4.9 (1.6)** hours, S (%) **73.9 (19.3)**

A2. Inclusion: ≥2 h of concordant sleep; sleep indices computed on total sleep.

ICU: 80 subjects (163 segments), TST **8.6 (3.0)** hours, S (%) **56.4 (18.7)**
Sleep lab: 190 subjects (190 segments), TST **5.3 (1.3)** hours, S (%) **76.7 (16.4)**

A3. Inclusion: ≥2 h of concordant sleep; sleep indices computed on concordant sleep.

ICU: 80 subjects (163 segments), CST **4.2 (1.7)** hours, S (%) **58.3 (26.9)**
Sleep lab: 190 subjects (190 segments), CST **4.1 (1.4)** hours, S (%) **76.8 (18.2)**

**FIGURE 2:**
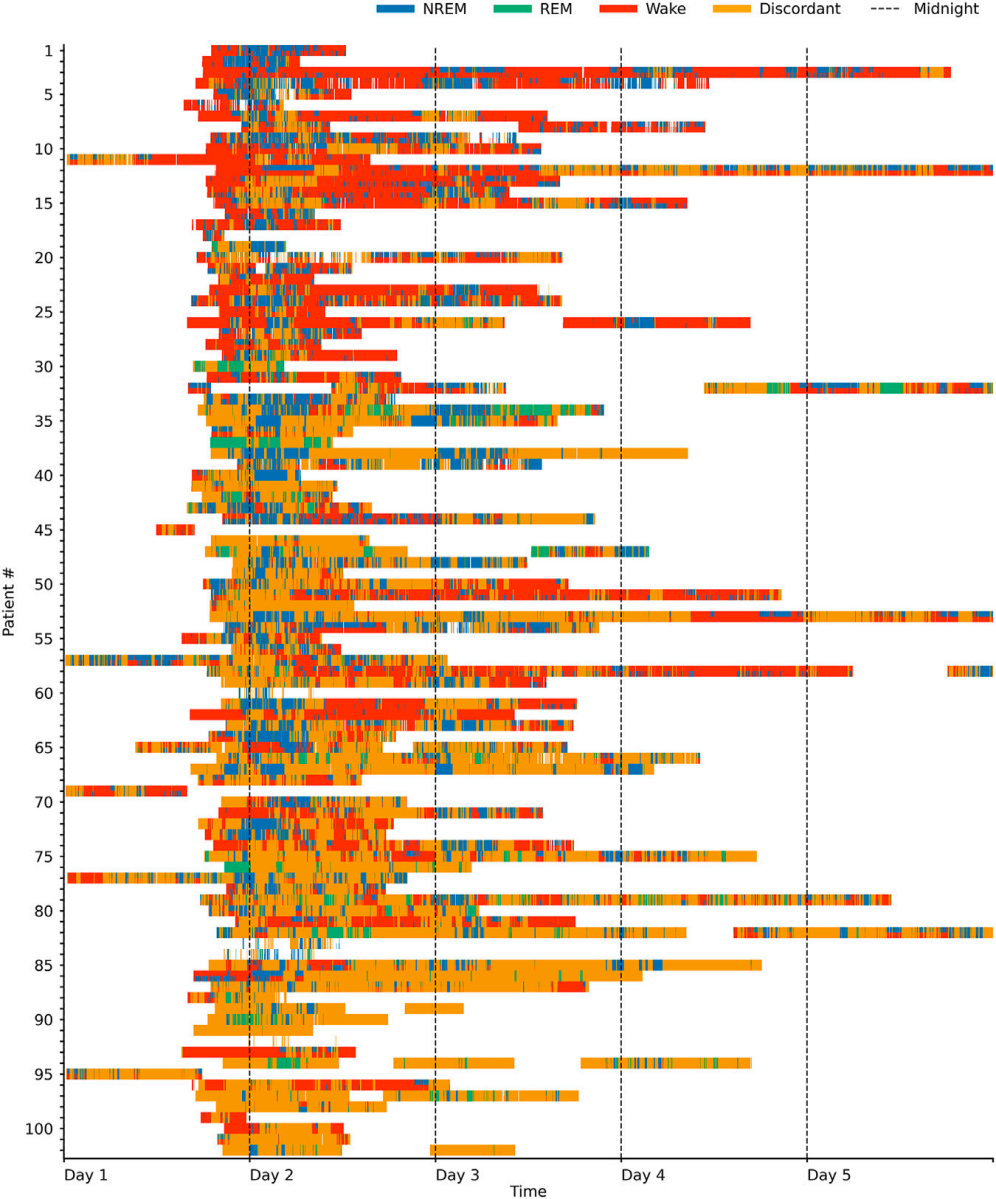
Sleep stages in the ICU over time. Swimmer plot visualizing sleep stages over time for 102 ICU patients. One line represents one patient, and patients are sorted by the proportion of sleep stage discordance (see main text for definition). For sleep epochs where HRV and breathing-based models were in concordance (60% of the data), the data is colored according to the sleep stages NREM (pooled N1, N2, and N3), REM and Wake as assigned by the breathing-based (top half of each line) and HRV-based (bottom half of each line) sleep staging models. Epochs where HRV and breathing-based models were discordant (40% of the data) are marked as orange. Both sleep stage distribution and the amount of discordance considerably varied among patients.

**FIGURE 3:**
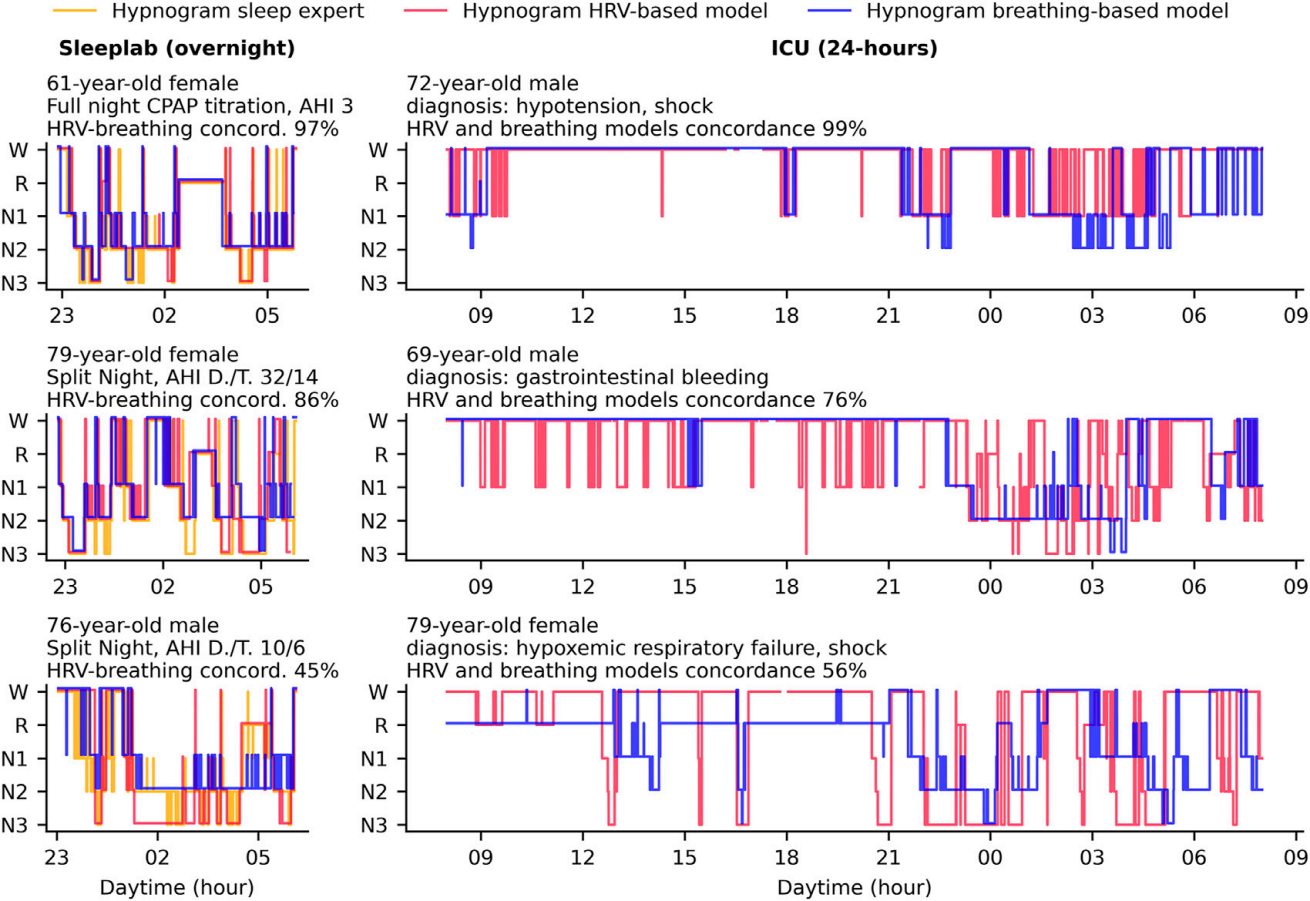
Hypnogram sample cases in the ICU and sleep laboratory. In this study, we used deep neural networks to stage sleep from both HRV-and breathing data, and defined concordance between the two resulting hypnograms (see main text). Hypnogram data for three sample patients from the sleep laboratory and three sample patients from the ICU are shown here and sorted by HRV- and breathing model concordance. The sleep laboratory data (clinical polysomnographies) included expert-scored sleep stages and respiratory events. Abbreviations: AHI D./T. Apnea-Hypopnea Index diagnostic/titration part of split night.

Sleep staging results for each patient over time are shown in Figure 2 and Supplementary Figure S2, sample hypnograms for both ICU and sleep lab patients are shown in Figure 3.

#### 3.3.1 Sleep indices

**FIGURE 4:**
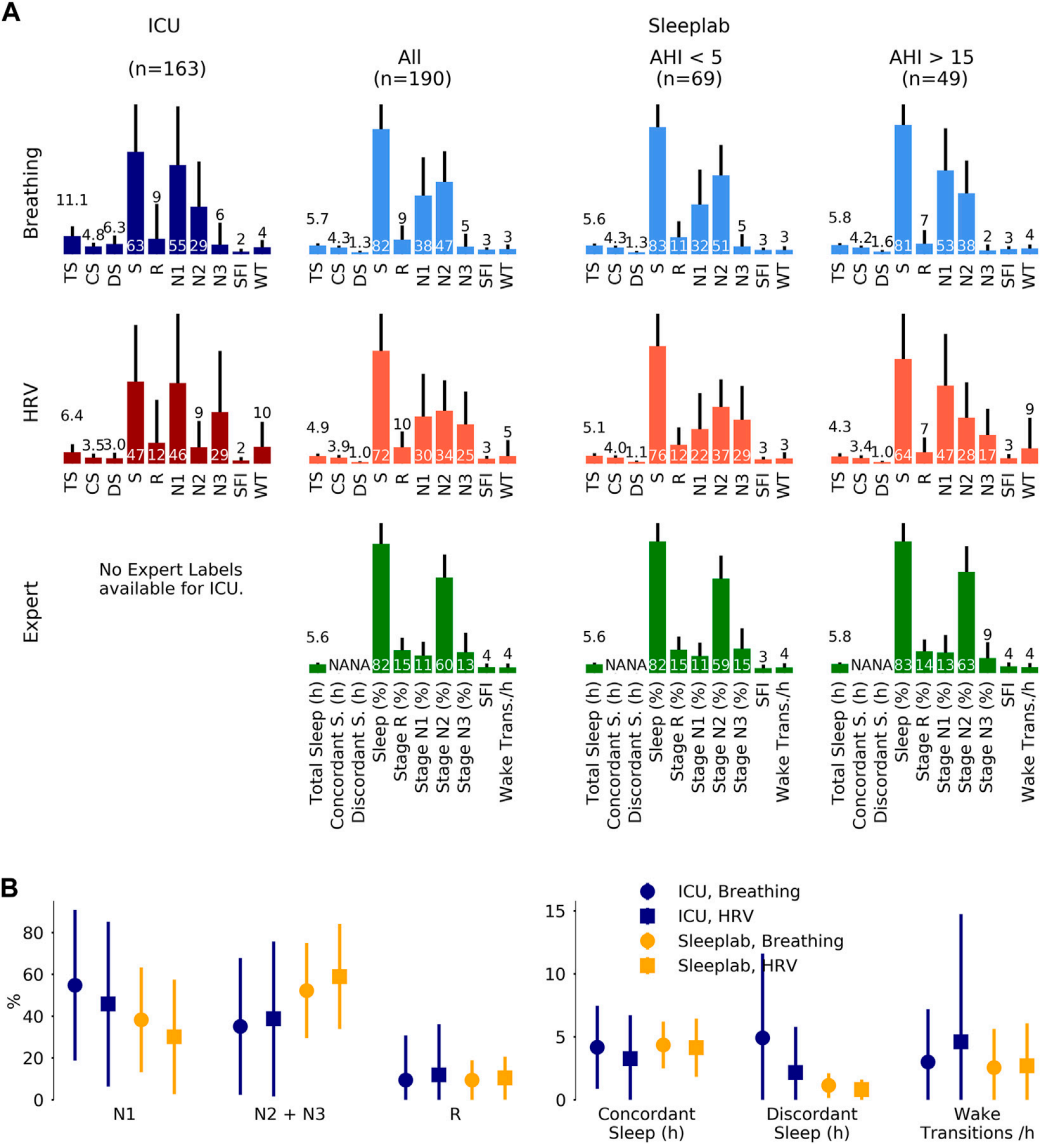
Sleep staging results. Sleep staging results for a surgical and medical ICU (N = 80 subjects, 163 24 h segments) and for an age- and sex-matched sleep laboratory cohort (N = 190 subjects, 190 nights), where for each 24 h segment or night sleep was detected at least once. Inclusion of all available ICU patients (N = 102), i.e., without minimum sleep requirement, resulted in a mean total sleep time of 6.2 (3.1) hours per 24 h in the ICU. Sleep stages were determined by breathing (respiratory effort) and heart rate variability (HRV)-based deep neural network models. For the sleep lab, additional human expert labels were available. **(A)**. Mean (one standard deviation) sleep indices. TS: total sleep time (hours), CS: concordant sleep time (hours), DS: discordant sleep time (hours), S: sleep percentage of total recording (%), SFI: sleep fragmentation index, WT: wake transitions per hour of sleep. **(B)**. Median (inter-quartile range) sleep indices for ICU and sleep lab cohort for both breathing- and HRV- based sleep staging models.

For approach A2 (see Methods), the results of the sleep indices computation on a 24 h level for the HRV and breathing models, together with the expert labels for the sleep lab data, are shown in Figure 4. Mean total sleep time per 24 h in the ICU was determined to be 6.5 h with the HRV-based model and 11.8 h with the breathing-based model (+81.5%). In the sleep lab, total sleep time was determined to be 4.9 h with HRV-based model and 5.7 h with breathing model (+16.3%) and 5.6 h by the human sleep expert.

**FIGURE 5:**
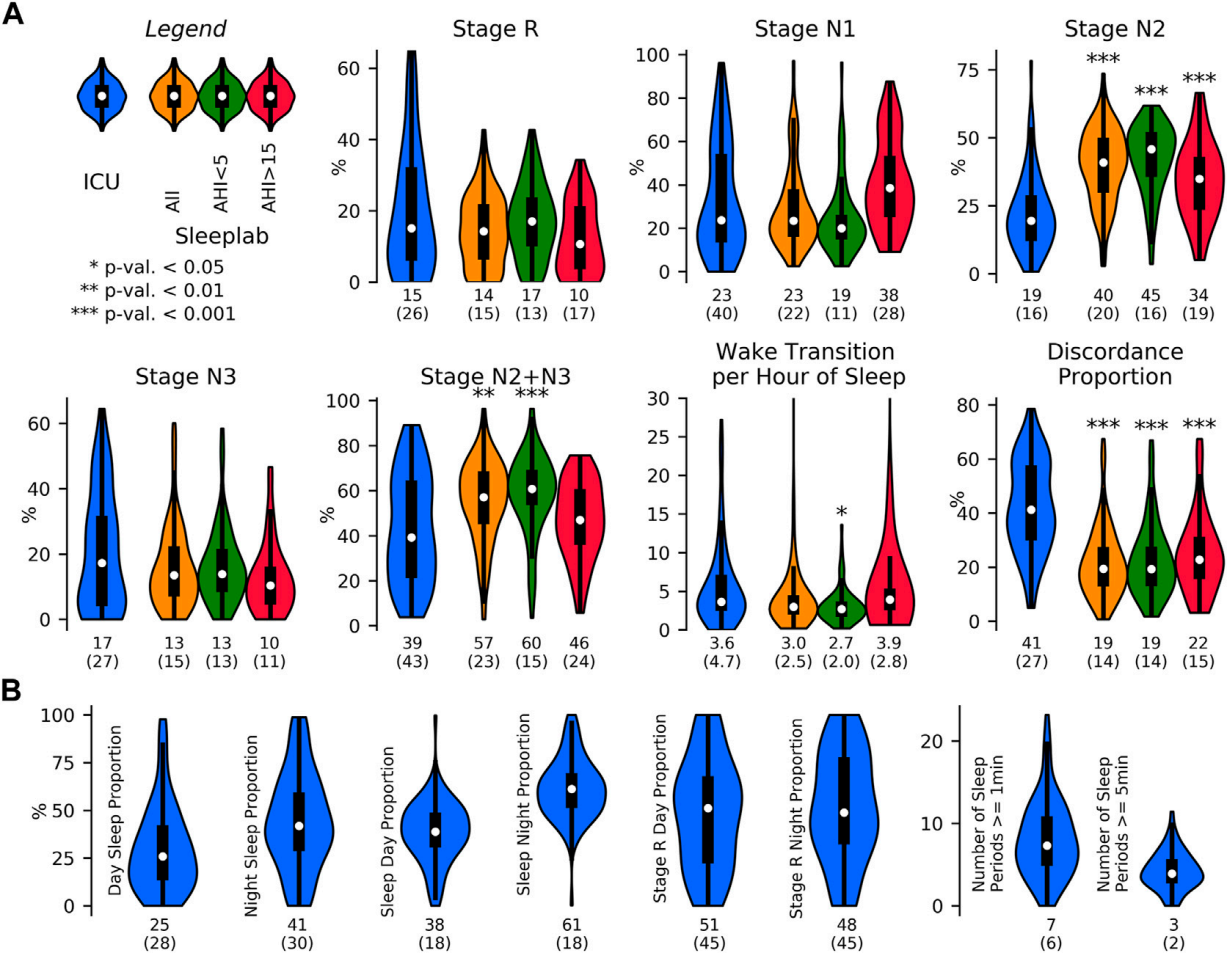
Sleep indices in the ICU and in the sleep laboratory. Sleep indices distribution visualized with violin plots and embedded boxplots (inter-quartile ranges: black rectangles) and medians (white dots); numerical values at bottom of distribution plots show median (interquartile range). **(A)**. Sleep Indices (mean of breathing and HRV-based indices) in the ICU (N = 80 patients) and age and sex-matched sleep lab cohorts (N all = 190 subjects, N AHI<5 = 69 subjects, N AHI>15 = 49 subjects), both with requirement of minimum of 2 h of detected sleep. Mann-Whitney U (MWU) tests and Mood’s median (MM) tests were applied to compare the ICU and sleep lab cohorts, and significance was indicated if both tests reached a given significance level. Stage R distributions showed heavier tails in the ICU cohort; statistical comparisons of differences did not reach significance. In the ICU, there was a larger proportion of light sleep (N1) compared to the AHI<5 sleep lab cohort (p MWU = 0.05), and significantly less N2 compared to all sleep lab cohorts. The proportion of N2+N3 was also reduced compared to total sleep lab and AHI<5 cohorts but not compared to subjects with AHI>15. Median wake transitions per hour of sleep were similar in the ICU and AHI>15 group (3.6 and 3.9), and significantly lower in the AHI<5 group (2.7). The ICU cohort showed a significantly greater proportion of discordance between the breathing and HRV-based sleep staging models. **(B)**. Sleep fragmentation indices obtained in the ICU. Median 25% of the day (08:00–20:00) and 41% of the night (20:00–08:00) were spent asleep. 38% of total sleep and 51% of R sleep occurred during the day.

Figure 5 depicts sleep indices (mean of breathing and HRV-based indices) distributions for all patients. In the ICU, there was a greater proportion of N1 compared to the AHI <5 sleep lab cohort (p MWU = 0.05) and significantly less N2 compared to all sleep lab cohorts (*p* < 0.001). The proportion of N2+N3 was reduced compared to total sleep lab (*p* < 0.01) and sleep lab AHI <5 (*p* < 0.001) cohorts but not compared to the sleep lab subgroup with AHI >15. While stage R distributions showed heavier tails in the ICU cohort (p MWU = 0.05). Median wake transitions per hour of sleep were similar in the ICU and sleep lab AHI >15 groups (3.6 and 3.9), but lower in the sleep lab AHI <5 group (2.7, *p* < 0.05). The ICU cohort showed a larger proportion of discordance between the breathing and HRV-based sleep staging models compared to the sleep lab cohort (41% and 19% respectively). In the ICU, a median of 25% of the day (08:00–20:00) and 41% of the night (20:00–08:00) were spent asleep. 38% of total sleep and 51% of R sleep occurred during daytime hours.

In post-hoc analyses, we investigated if the amount of N1, N2+N3, and the number of awakenings in the ICU is explained by the predictor variables age, sex, BMI, and SOFA score by fitting multivariable regression models. The total variance of N1 explained by the predictors was 6.4%, with no significant coefficients (age b = 0.4, *p* = 0.2; sex b = 9.2, *p* = 0.1; BMI b = 0.3, *p* = 0.6; SOFA b = 1.2, *p* = 0.5). The total variance of N2+N3 explained was 15.2% with significant associations with age and sex (age b = −0.6, *p* = 0.048; sex (with male as 1, female as 0) b = −11,6, *p* = 0.03, BMI b = 0.35, *p* = 0.5, SOFA b = −1.3, *p* = 0.4). The total variance of the number of awakenings was 4% with no significant associations. In an additional analysis, we have fitted a multilinear regression variable to the amount of REM sleep with predictor variables age, sex, BMI, SOFA, total amount of opioids, benzodiazepines and antipsychotics, and indicator variables for the primary diagnoses. The model explained 29% of the variance with no significant coefficients.

The following sleep indices results for the ICU were confirmed (same effect direction, all significant) by all analysis approaches A1-A3: Elevated discordant sleep time and proportion, reduced N2 proportion, and reduced N2+N3 proportion. The number of wake transitions per hour of sleep in the ICU was increased compared to the total sleep lab cohort (significant in 2 out of 3 analysis approaches), significantly increased compared to the sleep lab AHI <5 cohort (significant in all analysis approaches), and similar compared to the sleep lab AHI >15 cohort (not significantly different in any analysis approach). Results for the three analysis approaches are presented in Supplementary Tables S2–S6; a summary is presented in Supplementary Table S7.

#### 3.3.2 Subgroup analysis

**FIGURE 6:**
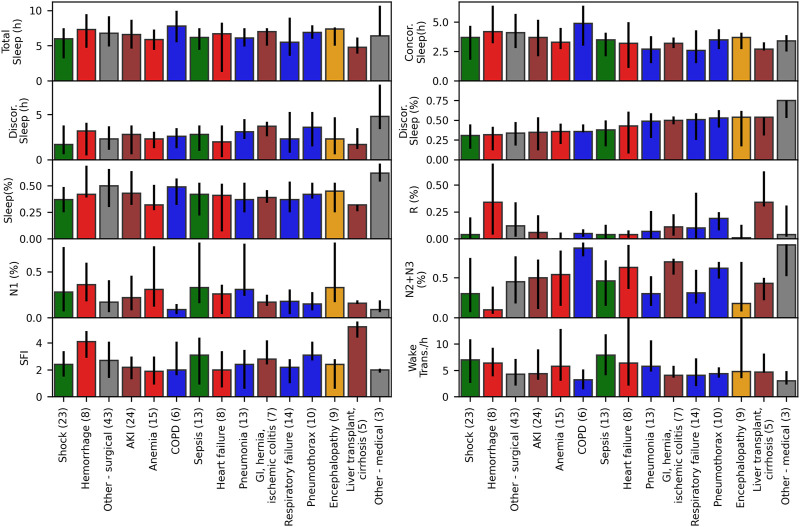
Sleep indices for ICU patients grouped by diagnosis. Median and interquartile ranges for sleep indices for ICU patients with at least 2 hours of detected sleep (N = 80), grouped by their primary or main condition, with number of patients per condition shown in parentheses. Kruskal Wallis tests were not significant (*p* > 0.05) for any of the sleep indices.

The median and interquartile ranges of sleep indices for patients grouped by diagnosis are shown in Figure 6 (and Supplementary Table S8 for numerical values including test results**)**. Kruskal Wallis tests were not significant for any of the sleep indices (minimum *p*-value of 0.08 observed for N3 proportion). Patients with lowest N2+N3 (%) observed had diagnoses of hemorrhage, shock, and encephalopathy, and patients with highest N1 (%) had diagnoses of hemorrhage, sepsis and encephalopathy. Discordant sleep proportion was highest for patients with cirrhosis and liver transplant, pneumothorax, respiratory failure, and pneumonia, and lowest for patients with shock, hemorrhage, and acute kidney failure.

#### 3.3.3 Latent feature representation of sleep

**FIGURE 7:**
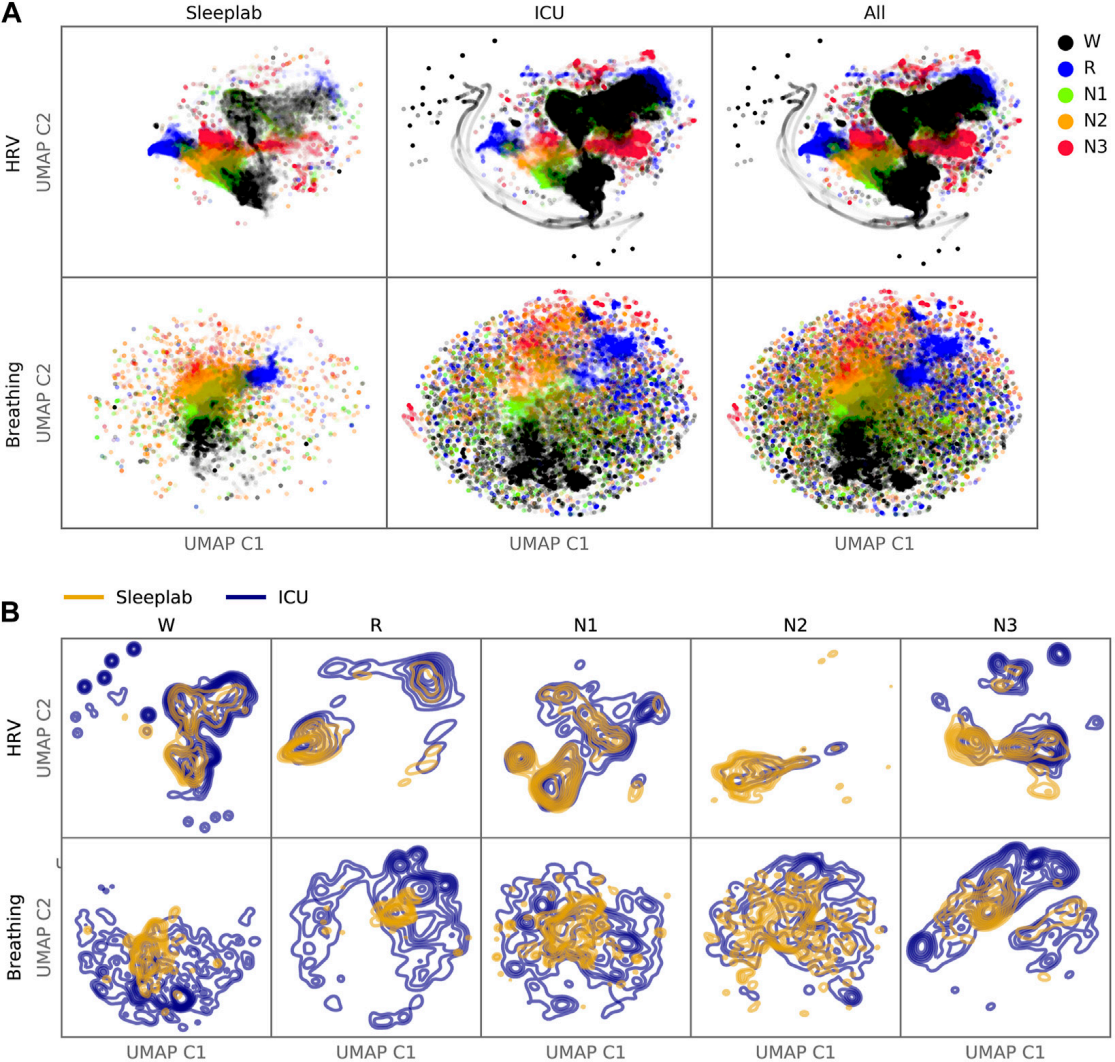
Latent Feature Analysis. Two-dimensional UMAP (Uniform Manifold Approximation and Projection) representations were computed with all epochs’ last hidden layer activations of the HRV-based and breathing-based deep neural networks. **(A)**. Data points are colored by predicted sleep stage, revealing clusters that correspond to sleep stages in both sleep lab and ICU data. **(B)**. For each sleep stage, probability density functions were estimated for the sleep lab and ICU data. While there is overlap between distributions (showing features computed from the ICU data are similar to features computed from the sleep lab data), the distributions of the ICU data are more dispersed (showing features in the ICU that are not present in the sleep lab data).

We generated two-dimensional maps by using the deep neural network last hidden layer’s activation for each epoch as inputs for the UMAP (Figure 7). Coloring the data points with the predicted sleep stage shows clusters that correspond to those sleep stages (Figure 7A). For some sleep stages more than one cluster is apparent (HRV: R, N1, N3; Breathing: R, N3). For each sleep stage, estimated Gaussian kernel densities of the UMAP-derived features show that the ICU features largely overlap with the sleep lab features, but are more widely dispersed (i.e., more variable) than in the sleep lab cohort (Figure 7B).

#### 3.3.4 Disagreement HRV and breathing model-error analysis

Out of 44 computed biosignal-based features, 27 showed a significantly different distribution (Mann–Whitney U test, significance level 0.01) between concordant and discordant sleep, see Supplementary Tables S9–S10 for details on the difference between HRV and breathing models. For N1, significantly reduced mean values in discordant sleep were observed for HRV-VLF, HRV-LF, HRV-HF, HRV-RMSSD, inter-breath-intervals, respiratory variability, ventilation CVar, CPC-LFC, CPC-HFC, and significantly increased for respiratory rate. For N2, NN-interval duration, inter-breath-intervals, respiratory variability, ventilation CVar were reduced, while HRV-VLF, HRV-LF, and respiratory rate were increased. For N3, NN-interval duration was decreased, while HRV-VLF, HRV-LF, HRV-HF, HRV-RMSSD, respiratory variability, ventilation CVar and CPC-LFC were increased. Lastly, for REM sleep, we observed increased HRV-RMSSD and CPC-HFC for discordant sleep compared to concordant equivalents.

In the multivariate linear regression analysis, the automated feature selection (see supplement for details) led to a model with 53 input variables (F-test statistic of 1.51, a F-test *p*-value of 0.036) and an r-squared value of 0.424. Hence, on a 24 h segment level, the computed HRV and breathing features could explain up to 42% of the variance of discordant sleep proportion.

None of SOFA scores, amounts of opioids, benzodiazepines or antipsychotics administered correlated with the discordant sleep proportion (all *p*-values for Pearson and Spearman correlations >0.05, see Supplementary Table S11).

### 3.4 Statistical analysis-Breathing

**FIGURE 8:**
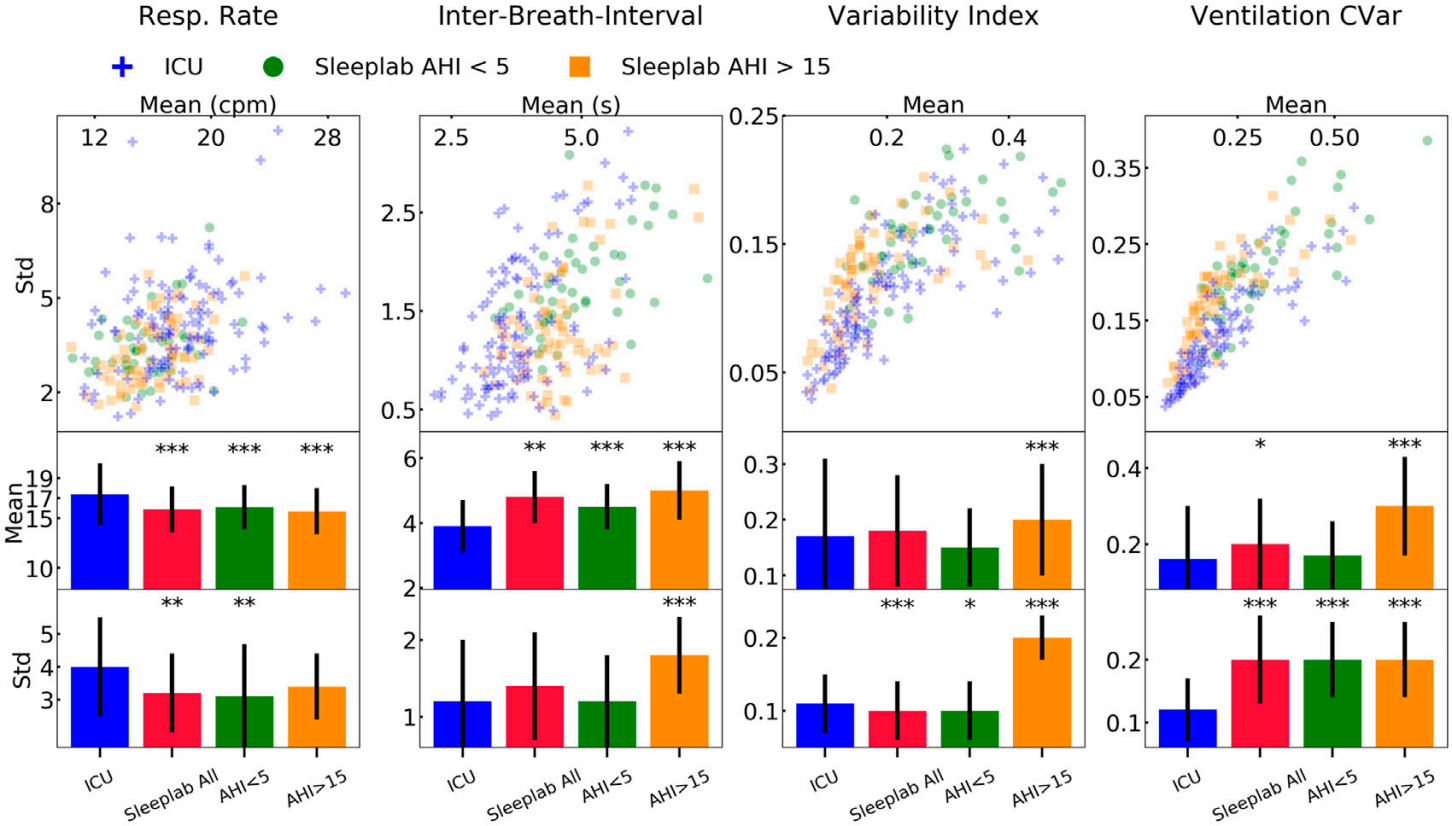
Results-breathing during sleep. Breathing features during sleep for ICU (N = 80) and sleep lab (N = 190) patients. From the respiratory effort breathing signal obtained with a wearable band, we computed four features for all patients: respiratory rate, inter-breath-intervals, variability index, and as a proxy for minute ventilation, ventilation coefficient of variation (CVar). We compared the Intensive Care Unit (ICU) cohort with the full sleep lab cohort and the Apnea-Hypopnea-Index (AHI)-stratified cohorts. Significance levels are denoted as * for *p* < 0.05 and *** for *p* < 0.001. We show the scatter plots stratified by each sleep stage and tables containing the numerical values in the Online Supplement.

Figure 8 shows the mean and standard deviation of breathing features during sleep for each patient. The mean and standard deviation of respiratory rate during sleep was significantly larger for the ICU (mean respiratory rates = 17.4 cycles per minute, respiratory rate standard deviation = 4 cycles per minute, *p* < 0.01), compared to the sleep lab patients. The median inter-breath-interval during sleep was significantly lower in the ICU (3.8 s) compared to the sleep lab (4.7 s). Effect directions were the same (with significance *p* < 0.001) for the sleep lab AHI <5 and sleep lab AHI >15 groups. The mean and standard deviations of the ventilation’s coefficient of variation (as a proxy for minute ventilation) was significantly smaller in the ICU (*p* < 0.05). The mean respiratory variability index in the ICU was smaller compared to the AHI>15 group (*p* < 0.001) and larger to the AHI<5 group (not significant). See Supplementary Table S12 for a summary and Supplementary Table S13 for details.

## 4 Discussion

Our main contributions are the following key findings: 1) In the ICU, heart rate variability (through ECG) and respiratory information can provide meaningful information about sleep stage, quality, and fragmentation-this is significant because HRV/respiratory signals are much more easily obtained in the ICU than conventional polysomnography and interactions within the sleep network are encoded within these signals; 2) Sleep stage concordance between HRV- and breathing-based models was associated with higher agreement with experts compared to sleep stage discordance; 3) The proportion of sleep stage concordance was lower in the ICU (60%) compared to the sleep laboratory cohort (81%); 4) Clear differences were evident between sleep indices of ICU patients and those of an age and sex-matched clinical sleep laboratory cohort, such as decreased N2 and N2+N3 proportions in the ICU; 5) Sleep indices of ICU patients, particularly awakening frequency, resemble those of sleep-disordered breathing (SDB) sleep laboratory patients more closely than those of non-SDB patients; 6) Sleep in the ICU is fragmented, consistent with a breakdown of the sleep network; 7) ICU patients showed higher respiratory rate compared to sleep laboratory patients, higher breathing variability compared to non-SDB sleep laboratory patients, and lower breathing variability compared to SDB sleep laboratory patients.

### 4.1 Sleep in the ICU

In this study we compared sleep of ICU patients with age and sex-matched sleep laboratory patients. Sleep indices of ICU patients obtained from the HRV- and breathing-based deep neural network sleep staging models were similar across three different sensitivity analyses. Compared to the sleep laboratory cohort, we observed a shift of NREM sleep stage proportion from N2 and N3 towards more light and non-restorative N1. This may be significant, as there has been growing evidence that deep NREM sleep has a crucial role in neurophysiological phenomena such as immunity, glucose metabolism, hormone release and memory (Léger et al., 2018). The median amount of REM sleep in ICU patients was lower compared to non-sleep disordered breathing and similar to sleep disordered breathing patients from the sleep laboratory. Further, sleep of ICU patients was highly fragmented: patients spent a median of 25% of the day and 41% of the night asleep. Moreover, 38% of total sleep and 51% of REM sleep occurred during the day.

The significant differences between the ICU and the sleep laboratory patients provide evidence that the sleep network is frequently deranged during critical care. Our results reinforce prior published findings (typically PSG studies with smaller sample size (Boyko et al., 2017)), including abnormal hypnograms, high arousal index, abnormal sleep stage shifts, a reduction of deep NREM and varying amount of REM sleep (Hilton, 1976; Aurell and Elmqvist, 1985; Freedman et al., 2001; Parthasarathy and Tobin, 2004; Elliott et al., 2013; Boyko et al., 2017). Furthermore, there is growing evidence that sleep in the ICU, especially for ventilated patients, is often abnormal and cannot be scored with traditional EEG-based scoring criteria (Cooper et al., 2000; Watson et al., 2013). Potentially, periods of discordance observed in our study coincide with EEG patterns that have been classified as “atypical sleep” and “pathological wakefulness” in previous studies examining sleep in the ICU(Cooper et al., 2000; Watson et al., 2013).

The presented results are important as there is evidence that sleep impairment in the ICU is associated with delirium (Watson et al., 2012). Delirium, a state of brain dysfunction with fluctuating awareness, disorganized thinking, and an altered level of consciousness (Watson et al., 2012), is associated with long-lasting cognitive impairment after hospital discharge and accelerated onset of dementia (Fong et al., 2015). While the mechanism of delirium is not entirely established, the absence or reduction of deep, restorative NREM sleep and REM sleep, and disturbances of circadian rhythms may be risk factors of developing delirium (Watson et al., 2012). Cardiovascular and pulmonary signals, as we have shown here, can help monitoring sleep more routinely in the ICU, which can guide measures to preserve sleep continuity, such as managing noise and light levels better or concentrating times of procedures and exams to allow more consolidated periods of rest.

### 4.2 Feasibility of staging sleep with heart rate variability and respiratory signals

The deep neural network activation patterns, visualized in 2D-space with embedding maps, of ICU patients shared similarities to those of sleep lab patients but additionally showed activation patterns uniquely observed in the ICU. This means that large parts of the ICU input data were processed by the artificial networks in such a way that the learned data representations (i.e., high-dimensional features about sleep before making the final sleep stage decision) resembled representations resulting from input data that are like the training data (the networks were trained using sleep lab data). This result helps mitigate concerns about a too-large covariance shift. Because the sleep network appears to be fundamentally changed in the ICU, activation patterns that are not present in sleep lab patterns are potentially of interest, and more research is needed to understand the significance and implications of these clusters, as they may affect reliability of models or indicate certain pathological states present in the ICU population and not in sleep laboratory populations. 27 out of 44 HRV, CPC, and breathing features significantly differed between concordant and discordant sleep epochs, indicating that these well-established and interpretable features might be used a-priori to assess the reliability in sleep stage assessment by HRV- and breathing-based sleep staging models. This result was confirmed with a multivariate linear regression model, using HRV and breathing features from 24 h segments as independent variables, where 42% of the variance of discordance proportion was explained by these features. Surprisingly, we found no association between the discordance of the models and use of opioids, benzodiazepines and antipsychotics, or health status of the patient (SOFA score). Similarly, patients grouped by primary and secondary diagnosis, did not show statistically significant differences in model discordance. This is remarkable, as we hypothesized more severe medical conditions would lead to a higher discordance, but non-significance may be a result of low sample size per subgroup.

These results demonstrate that the presented method of measuring sleep in ICU patients, insofar as our cohort is representative, is a potential alternative to EEG-based methods in many cases. We did observe more inconclusive sleep stage assessments (measured by discordance of the HRV- and breathing-based sleep staging models) than in the sleep laboratory. Although we found partial explanations for these discordances, more research is necessary to understand the critically-ill-specific relationships between brain and sleep states, HRV- and breathing signals.

### 4.3 Respiratory analysis

Mean respiratory rate during sleep was significantly higher in the ICU compared to all sleep lab cohorts. Variance of respiratory rate was significantly increased compared to total sleep lab cohort and AHI<5 subgroup but not compared to AHI>15 subgroup. Conversely, mean inter-breath-intervals were significantly lower in the ICU compared to all sleep lab cohorts. Interestingly, the variance of the inter-breath-intervals was also low in the ICU (comparable to sleep lab AHI<5, significantly lower than AHI>15 group). Breathing variability, which considers variability in timing and amplitude of breaths, was higher in the ICU group than in the AHI<5 sleep lab patients and lower than in AHI>15 sleep lab patients. This suggests that there is greater baseline instability of respiratory patterns in the ICU, likely reflecting acute illness, even when respiration is mildly abnormal. On the other hand, this level of instability is seen in healthier (non-ICU) patients with severe sleep apnea. Similarities of breathing statistics in ICU patients and in SDB sleep laboratory patients, and overall higher breathing variability in the ICU patients, underscore the high prevalence of sleep-disordered breathing in the ICU patients.

### 4.4 Limitations

In our observational study, we did not have EEG recordings available in the ICU, only in the sleep laboratory. Therefore, we could not analyze relationships of sleep EEG and the HRV- and breathing-based sleep stage assessments in ICU patients. Joint analysis of cerebral cortex, cardiac, and respiratory activity would allow a more holistic analysis and understanding of sleep physiology in the ICU but are a practical challenge in this population. The presented study could not and did not aim to investigate the question of how the presented non-EEG based sleep state estimation agrees with human sleep experts having EEG available. Rather, we aimed to investigate the properties and behavior of the different models (in the form of concordance) when used in critically ill patients, and with acknowledging the limitation of not having EEG but having a relatively large sample size, analyze sleep and estimate sleep indices. This may serve as a first step towards the goal of non-EEG based sleep staging in the ICU, and certainly studies need to confirm analyses with EEG measurements. Additionally, while this study focused on the assessment, analysis, and interpretation of sleep stages and sleep fragmentation, further research should also explore the potential of other non-EEG markers, such as cardiopulmonary coupling, as reliable tools for estimating sleep states in the ICU. Similarly, investigations should be carried out to determine the advantages of using ECG and respiration biosignals over actigraphy as markers for sleep states in the ICU.

Further limitations are that the training data for the models consisted of night-data only and potential circadian HRV variation over 24 h is therefore not accounted for. This unaccounted circadian effect is likely small compared to respiratory and pathological driving in ICU population. While the sample size of our study is relatively high compared with other sleep studies done in the ICU setting, 102 patients is still likely a relatively low number relative to the full spectrum of ICU patients and sleep physiology. As the target enrollment of the clinical trial is 450 patients, we hope to be able to follow up with more granular analysis of sleep in the ICU in the future. Patients in this cohort were admitted to surgical and medical ICUs, thus results in other types of ICU’s (e.g., neurological, cardiothoracic) might be different. Further, because we studied sleep in patients mostly not mechanically ventilated, the accuracy of the proposed sleep measurement method and reported sleep results during mechanical ventilation might differ. Finally, all patients were randomized into three groups as part of our clinical trial, where they were infused overnight for 11 h, starting usually at 8pm, with 0.1 or 0.3 mcg/kg/h dexmedetomidine (low dose) or placebo (normal saline). When this ongoing quadruple blinded trial is concluded, it will be possible to analyze the effects of low dose dexmedetomidine, if any, on our measures of interest.

We present the following hypotheses based on the outcomes of our observational study. Further research is needed to validate these findings: 1) Using ECG and respiration as biological signals can provide insight into sleep network pathologies in the ICU, though further research is needed to compare the accuracy to full polysomnography; 2) HRV and breathing patterns may contain information about disease pathologies in the ICU that is not captured by EEG analysis; 3) ICU patients may show reductions in signatures of deep NREM sleep, and dispersion of sleep into the day, compared to sleep laboratory cohorts; 4) The distribution of REM sleep among critically ill patients and within patients over time in the ICU may be uneven; and 5) The ability to monitor sleep-like states in the ICU without complex monitoring may enable better tracking of sleep-wake state boundaries and fragmentation, the impact of such fragmentation on delirium and other ICU outcomes, and even estimate the effects of sleep-targeted therapies.

## Data Availability

The datasets presented in this study can be found in online repositories. The names of the repository/repositories and accession number(s) can be found below: https://github.com/mghcdac/ecg_respiration_sleep_staging_icu.

## Abbreviations

NREM, non-rapid eye movement sleep; REM, rapid eye movement sleep; ECG, electrocardiogram; HRV, heart rate variability; ICU, intensive care unit, SOFA, sequential organ failure assessment; CCI, Charlson Comorbidity Index; TS, total sleep; CS, concordant sleep; DS, discordant sleep; S, sleep percentage; R, stage R; N1, stage N1; N2, stage N2; N3, stage N3; SFI, sleep fragmentation index; WT, wake transitions per hour of sleep; AHI, apnea hypopnea index; UMAP, Uniform Manifold Approximation and Projection; SDB, sleep disordered breathing; Std, standard deviation; IQR, inter quartile range; CVar, Coefficient of Variation.
