# Supplementary material for: Sleep staging in the ICU with heart rate variability and breathing signals. An exploratory cross-sectional study using deep neural networks
Source: Front Netw Physiol. 2023 Feb 27;3:1120390. doi: 10.3389/fnetp.2023.1120390 (PMC10013021; doi:10.3389/fnetp.2023.1120390)
Supplement: Supplementary file 1 [file Table1.DOCX]

Supplementary Material

**Sleep staging in the ICU with heart rate variability and breathing signals. An exploratory cross-sectional study using deep neural networks.**

Authors: Wolfgang Ganglberger, MS^1,2,3,6^, Parimala Velpula Krishnamurthy, MD^1,2^, Syed A. Quadri, MD^1,2^, Ryan A. Tesh, BS^1,2^, Abigail A. Bucklin, BA^1,2^, Noor Adra, BA^1,2^, Madalena Da Silva Cardoso, BA^1,2^, Michael J. Leone, MS^1,2,^, Aashritha Hemmige, MD^1,2^, Subapriya Rajan, MD^1,2^, Ezhil Panneerselvam, MD^1,2^, Luis Paixao, MD^1,2^, Jasmine Higgins, BA^1,2^, Muhammad Abubakar Ayub, MD^1,2^, Yu-Ping Shao, MS^1,2^, Brian Coughlin, BA^1^, Haoqi Sun, PhD^1,2,6^, Elissa M. Ye, MS^1,2^, Sydney S. Cash, MD, PhD^1,2^, B. Taylor Thompson, MD^4^, Oluwaseun Akeju, MD^5,6^, David Kuller, BS^7,^
Robert J. Thomas, MD^2,8*^, M. Brandon Westover, MD, PhD^1,2,6*^

Institutions:

^1^Department of Neurology, Massachusetts General Hospital (MGH), Boston, MA, USA

^2^Clinical Data Animation Center (CDAC), MGH, Boston, MA, USA

^3^Sleep & Health Zurich, University of Zurich, Zurich, Switzerland

^4^Department of Medicine, MGH, Boston, MA, USA

^5^Department of Anesthesia, Critical Care and Pain Medicine, MGH, Boston, MA, USA

^6^Henry and Allison McCance Center for Brain Health, MGH, Boston, MA, USA

^7^MyAir Inc., Boston, MA, USA

^8^Department of Medicine, Division of Pulmonary, Critical Care & Sleep, Beth Israel Deaconess

Medical Center, Boston, MA, USA

^*^Co-senior authors

Corresponding author:

M. Brandon Westover

Massachusetts General Hospital Neurology Department

55 Fruit Street

Boston, MA 02114

[mwestover@mgh.harvard.edu](mailto:mwestover@mgh.harvard.edu)

All authors have read and approved the submitted manuscript.

Data collection, data analysis and manuscript writing have been performed at Massachusetts General Hospital.

SUPPLEMENT MATERIAL

List of abbreviations:

ECG – electrocardiogram;
HRV – heart rate variability;
ICU – intensive care unit;
SOFA – sequential organ failure assessment;
CCI – Charlson Comorbidity Index;
TS – total sleep;
CS – concordant sleep;
DS – discordant sleep;
S – sleep percentage;
R – stage R;
N1 – stage N1;
N2 – stage N2;
N3 – stage N3;
SFI – sleep fragmentation index;
WT – wake transitions per hour of sleep;
AHI – apnea hypopnea index;
UMAP – Uniform Manifold Approximation and Projection;
SDB – sleep disordered breathing;
Std – standard deviation;
IQR – inter quartile range;
CVar – Coefficient of Variation;
p_dag – p-value D'Agostino's K-squared test;
p_sha – p-value Shapiro Wilk test;
p_tt – p-value Student’s t-test;
s_tt – t-statistic Student’s t-test;
p_mwu – p-value Mann Whitney U test;
s_mwu – Mann Whitney U statistic;
p_medians – p-value Mood’s median test;
s_medians – Mood’s Median test statistic.

*A. Dataset –* Patients’ eligibility for the clinical trial was determined according to the following inclusion/exclusion criteria taken from the Investigation of Sleep in the Intensive Care Unit^1^ ClinicalTrials.gov page:

Inclusion Criteria:

In order to be eligible to participate in this study, an individual must meet all of the following criteria:

1. Admitted to MGH Blake 7 or 12, or Ellison 4 ICU at Massachusetts General Hospital
2. Male or female, aged > 50 years.
3. Provision of signed and dated informed consent form (by patient or LAR).
4. Stated willingness to comply with all study procedures and availability for the duration of the study.
5. Not on mechanical ventilation at the time of enrollment.
6. Able to be enrolled before 7PM.
7. For females of reproductive potential: pregnancy test is negative.

Exclusion Criteria:

Any individual who meets any of the following criteria will be excluded from participation in this study:

1. Unable to be assessed for delirium (e.g. blindness or deafness).
2. Pregnancy or lactation.
3. Known allergic reactions to components of dexmedetomidine.
4. Follow-up would be difficult (e.g. active substance abuse, homelessness).
5. Severe dementia, as measured by a score of ≥3.3 on the Short Informant Questionnaire on Cognitive Decline in the Elderly (IQCODE).
6. Known pre-existing neurologic disease or injury with focal neurologic or cognitive deficits.
7. Serious cardiac disease (e.g. sick sinus syndrome, sinus bradycardia).
8. Severe liver dysfunction (Child-Pugh Class C).
9. Severe renal dysfunction (receiving dialysis).
10. Low likelihood of survival >24 hours.
11. Low likelihood of staying in the ICU overnight
12. Patient is receiving either of the anticholinergic drugs scopolamine or penehyclidine.
13. Concomitant enrollment in another study protocol that may interfere with data acquisition or reliability of measurements.
14. Deemed unsuitable for selection by the research team or ICU providers due to any medical, legal, social, or interpersonal issues that would either compromise the study or the routine care of patients.

**Table S1.** ICU and matched sleep laboratory cohort.

| Cohort | ICU | Sleeplab  All | Sleeplab  AHI < 5 | Sleeplab  AHI > 15 |
| --- | --- | --- | --- | --- |
| N Females | 41 | 98 | 40 | 18 |
| N Males | 62 | 122 | 37 | 34 |
| Ratio Males/Females | 1.51 | 1.24 | 0.93 | 1.89 |
| Median Age  [IQR] | 68 [62.5, 75.0] | 67.3  [60.6, 73.4] | 66.0  [59.9, 69.5] | 70.5  [60.5, 74.8] |
| t-test Welch  p-value | - | 0.78 | 0.14 | 0.51 |
| Mann-Whitney U test p-value | - | 0.78 | 0.15 | 0.49 |
| Median Age Female [IQR] | 67 [57, 76] | 66.2  [59.9, 71.9] | 65.5  [58.8, 69.8] | 71.8  [67.3, 76.1] |
| t-test Welch p-value | - | 0.88 | 0.36 | 0.19 |
| Mann-Whitney U test p-value | - | 0.87 | 0.36 | 0.15 |
| Median Age Male [IQR] | 68 [64, 75] | 68.3  [61.8, 74.2] | 66.9  [62.1, 69.5] | 69.7  [60.1, 74.0] |
| t-test Welch p-value | - | 0.92 | 0.41 | 0.56 |
| Mann-Whitney U test p-value | - | 0.92 | 0.40 | 0.56 |


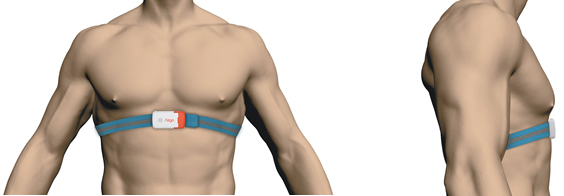


**Figure S1.** The wearable respiration device (‘Airgo’, MyAir LLC, Boston) used in this study.

1. *Biosignals Preprocessing*

For ECG analysis, we mainly used the open source ‘PhysioNet Cardiovascular Signal Toolbox’ ^2^. The settings below correspond to the toolbox parameters we used for this study.

Window Size: 300 seconds windows; increment 30 seconds; minimum of 20% of data needs to be good quality for a window to be considered for analysis; maximum 15% of data is allowed to be missing in a window in order to be considered for analysis.

Signal Quality: low quality threshold = 0.9, comparison window length = 10 seconds; increment = 1 second; time threshold = 0.1 seconds; Margin time not to include in comparison = 2 seconds.

Preprocessing: Maximum believable gap in RR intervals = 2 seconds, Percent limit of change from one interval to the next = 100%; outlier method = ‘remove outlier points’, signal quality threshold for good data = 0.9; minimum length of good data = 30 seconds.

Frequency Domain Analysis Settings: ULF = [0 .0033]; VLF = [0.0033 .04]; LF = [.04 .15]; HF = [0.15 0.4]; Power Spectral Estimation Technique: Lomb Scargle.

Peak Detection Settings: Refectory period = 0.2 seconds; energy threshold = 0.15, window size for QRS detection = 15 seconds.

1. *Sleep Staging*


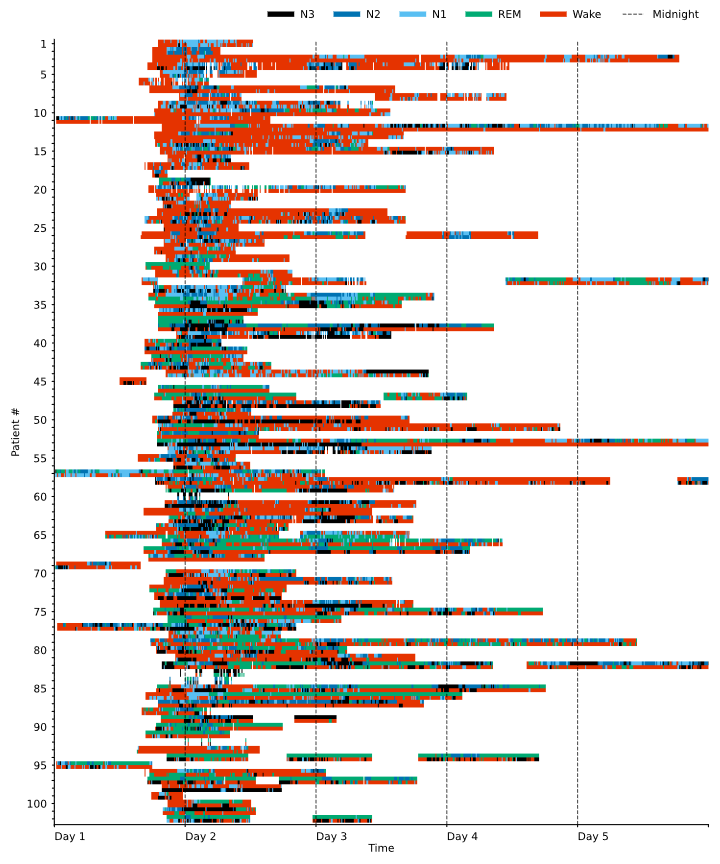


**Figure S2.** Swimmer plot visualizing sleep stages over time for 102 ICU patients. One line represents one patient and patients are sorted by the proportion of sleep stage discordance. The data is colored according to the sleep stages N1, N2, N3, REM and Wake as assigned by the breathing-based (top half of each line) and HRV-based (bottom half of each line) sleep staging models. Both sleep stage distribution and the amount of discordance considerably varied among patients.


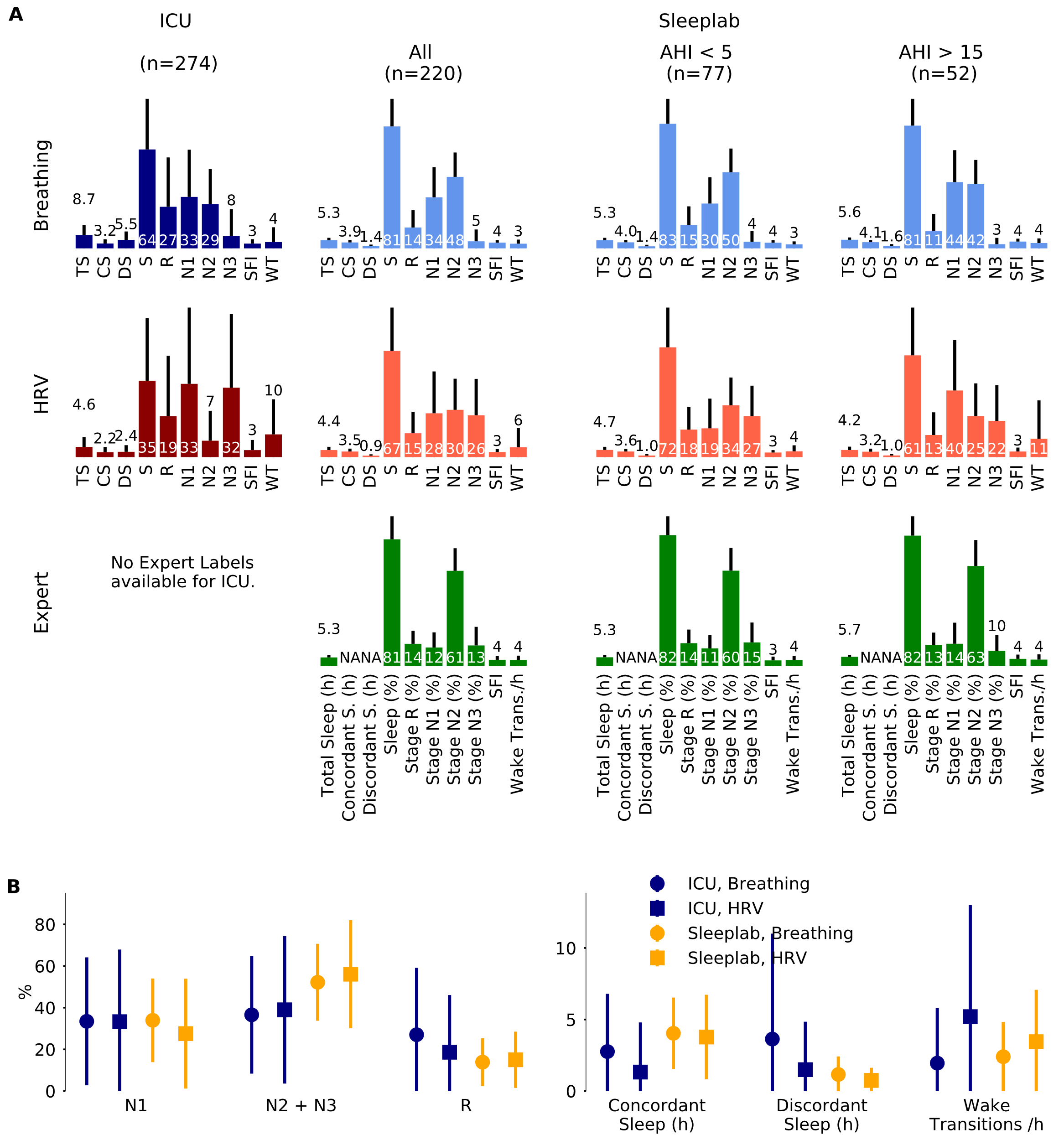


**Figure S3.** Analysis Approach A1. Sleep staging results for a surgical and medical ICU (N=102 subjects, 274 24-hour segments) and for an age and sex matched sleeplab cohort (N=220 subjects, 220 nights). Segments inclusion criteria: any sleep, sleep indices computed on total sleep. Sleep stages were determined by breathing (respiratory effort) and heart rate variability (HRV)-based deep neural network models, for the sleeplab additional human expert labels were available. **A.** Mean (one standard deviation) sleep indices for breathing, HRV and expert-based sleep stages (rows), and ICU, sleeplab and Apnea-Hypopnea Index (AHI) subgroups (columns). TS: total sleep time (hours), CS: concordant sleep time (hours), DS: discordant sleep time (hours), S: Sleep percentage of total recording (%), SFI: sleep fragmentation index, WT: wake transitions per hour of sleep. **B.** Median (inter-quartile range) sleep indices for ICU and sleeplab cohort for both breathing and HRV based sleep staging models.


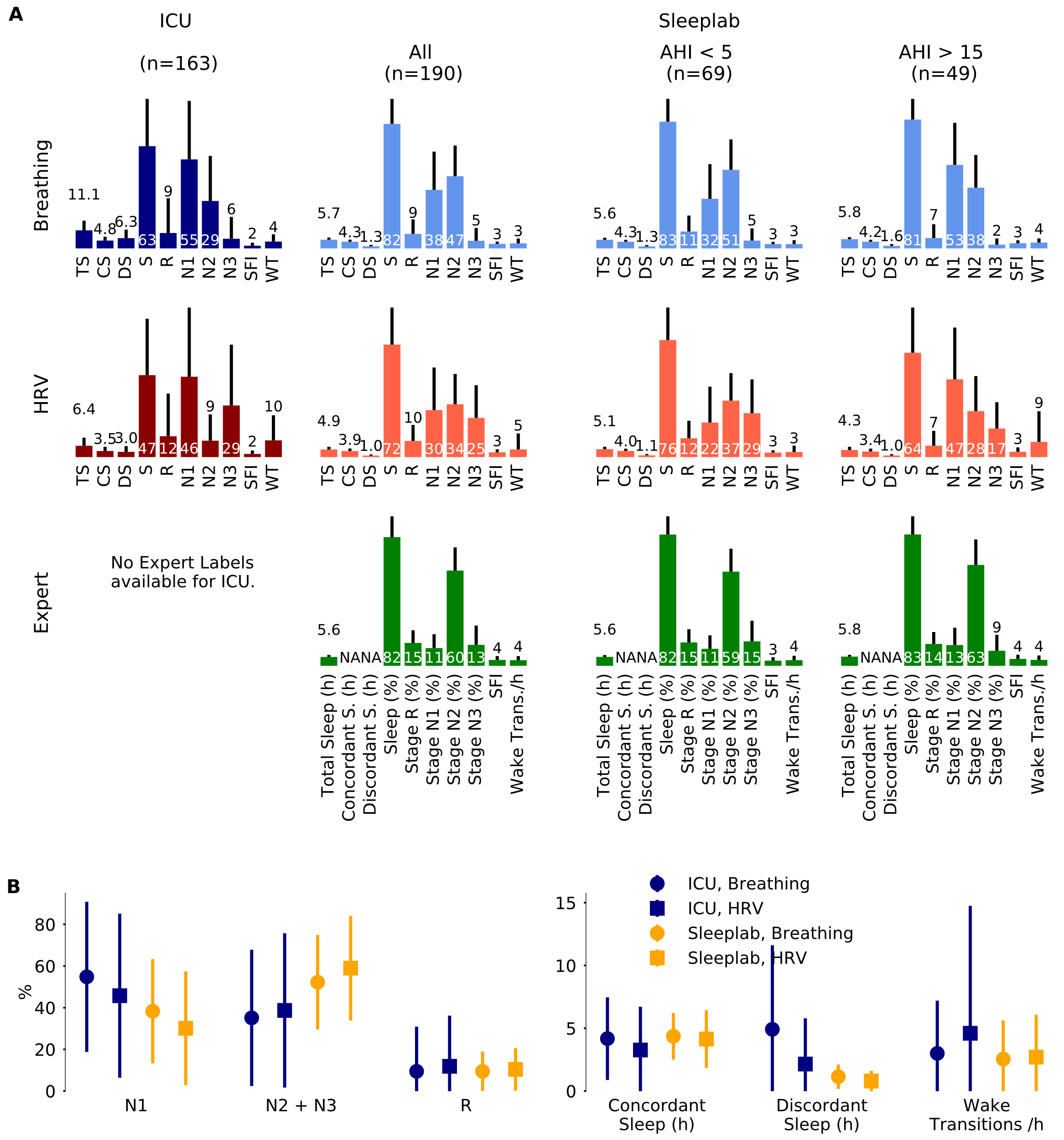


**Figure S4.** Analysis Approach A3. Sleep staging results for a surgical and medical ICU (N=80 subjects, 163 24-hour segments) and for an age and sex matched sleeplab cohort (N=190 subjects, 190 nights). Segments inclusion criteria: at least two hours of concordant sleep, sleep indices computed on concordant sleep. Sleep stages were determined by breathing (respiratory effort) and heart rate variability (HRV)-based deep neural network models, for the sleeplab additional human expert labels were available. **A.** Mean (one standard deviation) sleep indices for breathing, HRV and expert-based sleep stages (rows), and ICU, sleeplab and Apnea-Hypopnea Index (AHI) subgroups (columns). TS: total sleep time (hours), CS: concordant sleep time (hours), DS: discordant sleep time (hours), S: Sleep Percentage of total recording (%), SFI: sleep fragmentation index, WT: wake transitions per hour of sleep. **B.** Median (inter-quartile range) sleep indices for ICU and sleeplab cohort for both breathing and HRV based sleep staging models.

Wake and N1 predictions in the ICU data:
Out of all epochs that were

1.) Scored as N1 by breathing (N=50,536): 92% were scored as either N1 or W by HRV model (18% N1, 75% W).

2.) Scored as W by breathing (N=118,569): 99% were scored as either N1 or W by HRV model (88% W, 10% N1).

3.) Scored as N1 by HRV (N=27,972): 78% were scored as either N1 or W by breathing model (32% N1, 45% W).

4.) Scored as W by HRV (N=147,821): 97% were scored as either N1 or W by breathing model (71% W, 26% N1).

Wake and N1 predictions from HRV and breathing-based models agreed to a large extent, despite expected increased sleep-wake transition instabilities in the ICU.

**Table S2.** Analysis approach A1. Summary of sleep stage analysis with average HRV and breathing-based sleep stage assessments. Inclusion all segments with any sleep, sleep statistics computed on total sleep.

**Table S3.** Analysis approach A2. Summary of sleep stage analysis with average HRV and breathing-based sleep stage assessments. Inclusion > 2 hours of concordant sleep, sleep statistics computed on total sleep.

**Table S4.** Analysis approach A3. Summary of sleep stage analysis with average HRV and breathing-based sleep stage assessments. Inclusion > 2 hours of concordant sleep, sleep statistics computed on concordant sleep.

**Table S5.** Analysis approach A2. Main summary of sleep stage analysis with average HRV and breathing-based sleep stage assessments.

**Table S6.** Analysis approach A3. Main summary of sleep stage analysis with average HRV and breathing-based sleep stage assessments.

**Table S7.** Summary and remarks interpretation for sleep stage analysis for ICU and sleeplab comparison.

| Result | **Analysis Approach A2.** Inclusion >2h conc., statistics on total sleep | **Analysis Approach A3.** Inclusion >2h conc.,  statistics on concordant sleep | **Analysis Approach A1.** Inclusion any sleep,  Statistics on total sleep | Remark |
| --- | --- | --- | --- | --- |
| Data for analysis | ICU: 80 subjects, 163 segments.  Sleeplab: 190 subjects, 190 segments | ICU: 80 subjects, 163 segments.  Sleeplab: 190 subjects, 190 segments | ICU: 102 subjects, 274 segments.  Sleeplab: 220 subjects, 220 segments |  |
| Elevated median total sleep time (concordant + discordant sleep) for ICU compared sleeplab All group | Yes  [8.2 vs 5.5] (p<0.0001) | Not applicable (same total sleep time as column 1) | Yes  [6.0 vs 5.2] |  |
| Reduced concordant sleep time | Not significant  [4.0 vs. 4.2] | Same as column 1. | Yes  [2.3 vs. 3.9]  (p<0.0001) | 1. A1 contains all ICU data with any sleep. Therefore, median concordant sleep is lower for A1. If only ICU patients with >2 hours of concordant sleep are included, amount of concordant sleep is similar to sleeplab. |
| Elevated discordant sleep time (hours) | Yes [3.9 vs. 1.0] (p<0.0001) | Same as column 1. | Yes [3.4 vs. 1.0]  (p<0.0001) | For all data selection options, we observe significantly elevated discordant sleep time. |
| Elevated fraction discordant sleep of total sleep (%) | Yes [41.2 vs. 19.4, p<0.0001] | Same as column 1. | Yes [48.2 vs. 20.6, p<0.0001] | For all data selection options, we observe significantly elevated discordant sleep fraction. |
| Decreased “sleep efficiency” (%) (= time spent asleep / data available) | Yes  [58.2 vs. 81.1]  (p<0.0001) | Yes [54.8 vs. 82]  (p<0.0001) | Yes  [51 vs. 80]  (p<0.0001) | Not comparable, as patients in sleeplab are only there during night for sleep and patients spend full day in ICU. Amount of data available per patient in the ICU varies. |
| % REM sleep | Similar Median [15.1 vs. 14.2, n.s.] and higher mean [21.9 vs. 14.7, p<0.01] | Lower Median [0 vs. 7.8, p<0.001],  Higher mean (12.1 vs. 9.9, n.s.) | Higher Median [17.4 vs. 13.8, n.s.] and higher mean [23.4 vs. 14.4, p<0.0001] | Mixed results for different variants. Generally, the REM% distribution has a long right tail, resulting in larger mean than median. |
| % N1 sleep | Similar Median [23.7 vs. 23.5, n.s.],  Larger mean [33.5 vs. 29.5, n.s.] | Larger Median [37.6 vs. 25.7, p=0.05],  Larger mean  [46.2 vs. 34.2, p<0.01] | Larger Median [29.2 vs. 24.1, n.s.],  Larger mean [32.5 vs. 30.7, n.s.] | Indication of increased N1% in ICU compared to all sleeplab patients. ICU N1% is elevated compared to AHI<5 group in all 3 versions, and is reduced compared to AHI>15 in all 3 versions. |
| % N2 sleep | Reduced Median [19.5 vs. 40.9, p<0.0001],  Reduced mean [22.2, 39.7, p<0.0001] | Reduced Median [19.3 vs. 43.6, p<0.0001],  Reduced mean [21.5 vs. 40.6, p<0.0001] | Reduced Median [17.5 vs. 40.4, p<0.0001],  Reduced mean [18 vs. 38.6, p<0.0001] | In all 3 versions, N2 sleep is not only reduced compared to all sleeplab groups but also compared to AHI<5 and AHI>15 group. |
| % N3 sleep | Increased Median [17.2 vs. 13.5, n.s.],  Increased mean [21 vs. 15.8, p<0.05] | Reduced Median [11.1 vs. 12.5, n.s.],  Increased mean [18.8 vs. 15, n.s.] | Increased Median [15.3 vs. 12.8, n.s.],  Increased mean [20 vs. 15.5, p<0.01] | None of the results reaches significance level of 0.05. Using concordant sleep for analysis only (A3) decreases amount of N3% in ICU (15.3 to 10.3). Hypothesis: non-concordant N3 sleep contains pathological states that ‘look like N3’ in either N3 or breathing domain, but not both. |
| % (N2 + N3) sleep | Reduced Median [39.3 vs. 57.1, p=0.05],  Reduced mean [43.2 vs. 55.5, p<0.0001] | Reduced Median [33.6 vs. 58.7, p<0.01],  Reduced mean [40.2 vs. 55.6, p<0.001] | Reduced Median [35.3 vs. 55.8, p<0.01],  Reduced mean [38 vs. 54.1, p<0.0001] | In all 3 versions, N2+N3 sleep is reduced to total sleep lab group and AHI>15 group. |
| Increased Wake Transition/hour of sleep | Similar median [3.6 vs. 3] | Increased median [4.1 vs. 2.6, p<0.01] | Increased median [5.3 vs. 3.1, p<0.01] | Indication of increased number of wake transition per hour of sleep in the ICU. Significantly increased compare to AHI<5 groups in all three analysis approaches. |

*Latent feature representation of sleep*

The last hidden layers’ dimensions (D_LHL) for the Long Short-Term Memory (LSTM) networks were (40, 1) for the HRV-based model and (200, 1) for the breathing-based model. We performed two UMAPs (Uniform Manifold Approximation and Projection) separately for the data resulting from the HRV-based and breathing-based models. The python package UMAP^3^ was used to perform the UMAPs, and python packages matplotlib and seaborn were used for visualization.

Pseudo code:

1. Select either HRV-based or breathing-based model, and concatenate all last hidden layer activations for both ICU and sleeplab data, resulting in an array (‘LHL array’) with shape:
   (N_ALL, D_LHL) with N_ALL = 693,401, number of epochs from sleeplab data (324,928) plus number of epochs from ICU data (368,473), and D_LHL=40 for HRV- and D_LHL=200 for breathing-based model.
2. Perform unsupervised UMAP with input LHL array and select the first two dimensions of the resulting UMAP embedding, resulting in an array (N_ALL, 2). Parameters: number of neighbors=15, minimum distance=0.1, metric = “euclidean”.
3. For **Figure 3A**, plot UMAP embeddings for sleeplab data only, ICU data only, and sleeplab and ICU data combined (three figure panel columns). For each plot, color the data according to the assigned sleep stage.
4. For **Figure 3B**, for each panel column, select a sleep stage (W, R, N1, N2, N3) and estimate kernel densities for the UMAP embeddings of both the sleeplab and ICU data. Plot the 10% iso-proportion levels.

*Disagreement HRV and breathing model - error analysis.*

In order to identify features in the cardiovascular and pulmonary system that are associated with discordant sleep, we compute breathing and heart rate variability related features and compare the values of those features for agreeing and discordant sleep. We use the four breathing features as described above: respiratory rate, inter-breath-intervals, variability index, and ventilation CVar. For HRV, we compute a root mean squared successive difference of NN intervals (RMSSD), very low frequency (VLF, 0.0033-0.04 Hz), low frequency (0.04-0.15 Hz), and high frequency (HF, 0.15-0.4 Hz) with a 5-minute sliding window, using Lomb-Scargle periodogram for spectral analysis. We further compute Cardiopulmonary Coupling (CPC)^4^, a frequency domain analysis method that combines cross spectral power and coherence of the respiratory and HRV signals. Unlike in the original publication where respiratory signal is estimated from the ECG signal^4^, we use the wearable respiratory signal. From the CPC spectrogram, we compute the amounts of low frequency coupling (LFC, 0.01-0.1 Hz) and high frequency coupling (HFC, 0.1-0.4 Hz) with an 8.5-minute sliding window.

Summary features computed:

1. Heart Rate Variability: NN interval, mean very low frequency power (VLF, 0.0033-0.04 Hz), low frequency power (0.04-0.15 Hz), high frequency power (HF, 0.15-0.4 Hz), RMSSD of NN intervals.
2. Breathing: inter-breath-interval, respiratory rate, variability index, ventilation coefficient of variation.
3. Cardiopulmonary Coupling: low frequency coupling (LFC, 0.01-0.1 Hz), high frequency coupling (HFC, 0.1-0.4 Hz).

We computed the mean feature values (e.g. mean NN interval) for concordant and discordant parts for every 24-hour segment (inclusion criteria: at least 2 hours of concordant sleep) and performed a Wilcoxon signed-rank test with an alpha value of 0.01 for each feature pair. Features were computed for each sleep stage. Note: Discordant sleep is partially asymmetric, an epoch that is classified as N2 by model A and as Wake by model B is included for model A analysis here but not for model B analysis. Further, if model A classifies an epoch as N1, and model B as N3, the epoch is treated as ‘N1-discordant’ for model A and ‘N3-discordant’ for model B.

**Table S8**. Median and interquartile ranges for sleep indices for ICU patients grouped by their primary or main condition. Data is graphically displayed in Figure 6.

**Table S9.** Error Analysis – Details. HRV and respiratory features (rows in table) were computed for concordant sleep and discordant sleep, for both the HRV-based and breathing-based sleep staging models, and for each sleep stage. Columns contain number of 24-hour segments available after inclusion criteria (minimum 2 hours of concordant sleep), feature values, and the Mann–Whitney U test (both test statistic and p-value). The ‘significance’ column shows ‘True’ if the p-value is less than 0.01 for any of the HRV- or breathing-based sleep staging models.

**Table S10.** Shows the significant effect directions of Table S9 for easier readability. I.e. ‘+’/’-‘ indicate a significant (0.01 level) increase/decrease of a feature in discordant sleep compared to concordant sleep.

For each 24-hour segment included (inclusion criteria: at least 2 hours of concordant sleep), we obtained the proportion of discordant sleep, which is a number in the [0, 1] interval. To make the variable unbounded and more gaussian, we used the following transformation:

*proportion_transformed = ln (proportion / (1 – proportion)).*

We computed the mean, standard deviation, 0.25-, 0.50-, and 0.75 quantiles for each feature for every 24-hour segment and for each sleep stage. This lead to 886 features, and as a consequence, we used the LASSO penalty for multilinear regression. We start with a penalty of 0 and with a step size of 0.1, we increase the penalty until 10. For each penalty value, we train a LASSO model, obtain the variables included in the model and with those variables train a vanilla multilinear regression model for which we obtain the F-test statistic, F-test p-value and the r-squared. The result is shown in **Figure S4**, indicating overfitted models close to a penalty of 0 and underfitted models close to a penalty of 10. A penalty of 4 results in a regression model that contains 53 variables, an F-statistic of 1.51, a F-test p-value of 0.036, and an r-squared of 0.42. Hence, we obtained evidence that up to 42% of the variance in the discordance sleep proportion over a full 24-hour segment can be explained by HRV and breathing based features that are computed over pooled discordant and concordant parts.


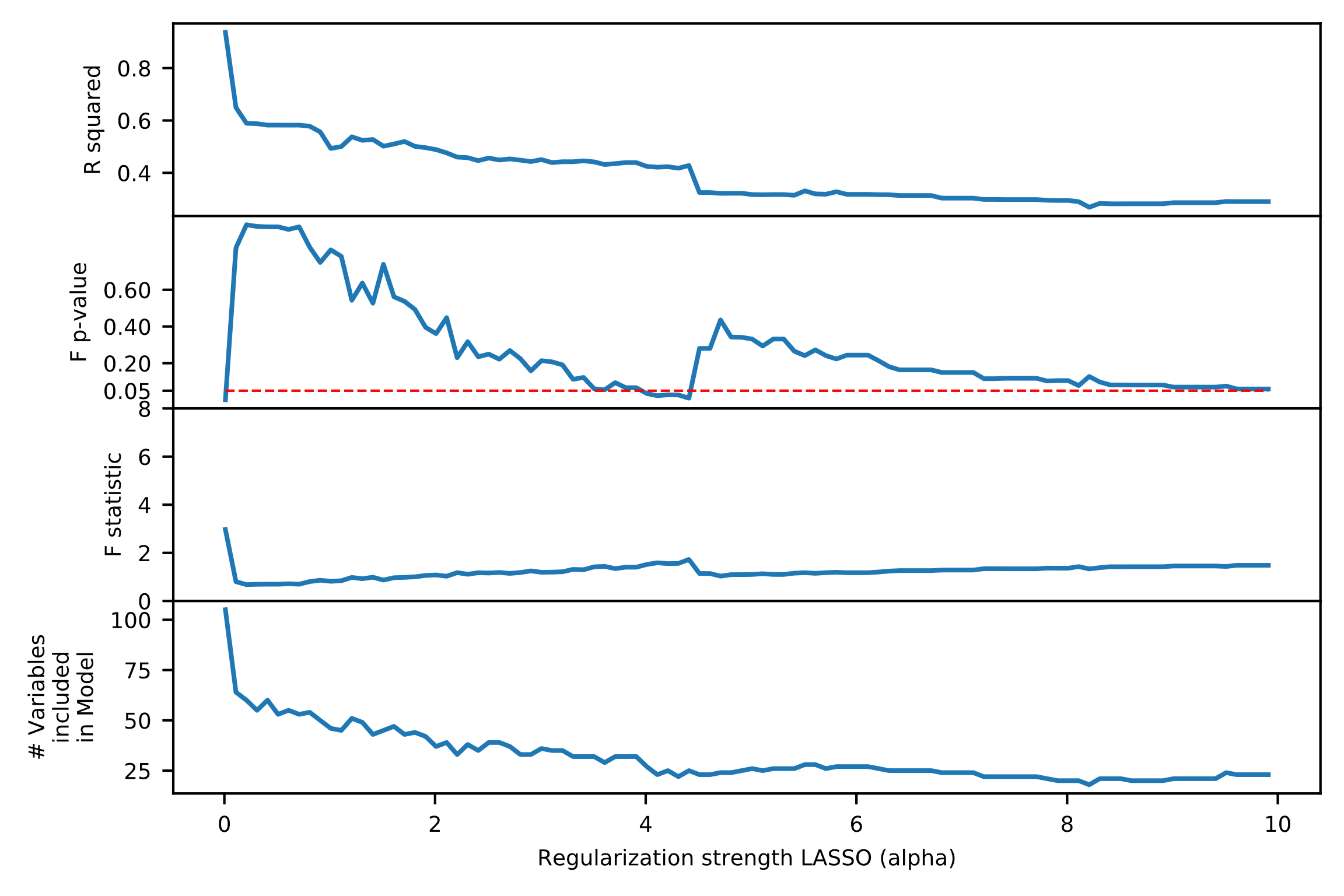


**Figure S5.** Model development for error analysis. We trained linear regression models with varying LASSO penalty. A penalty of 4 results in a regression model that contains 53 variables, an F-statistic of 1.51, a F-test p-value of 0.036, and an r-squared of 0.42, showing 42% of the variance in discordant sleep proportion is explained by this model.

**Table S11**. Correlations between proportion of discordant sleep and Sequential Organ Failure Assessment (SOFA) score or medications administered. Medications included in the analysis: Opioids (Buprenorphine, Morphine, Oxycodone, Hydrocodone, Hydromorphone, Fentanyl, Meperidine, Codeine, Tramadol), Benzodiazepines (Alprazolam, Chlordiazepoxide, Clonazepam, Diazepam, Lorazepam, Midazolam, Oxazepam, Phenobarbital, Propofol), Antipsychotics (Olanzapine, Clozapine, Thiothixene, Haloperidol, Fluphenazine, Prochlorperazine, Trifluoperazine, Loxapine, Quetiapine, Asenapine). Medications within a category were converted to equivalent doses before summed^5–7^.

*F. Statistical Analysis – Breathing.*

**Table S12.** Breathing Main Summary. Mean feature per night distributions.

**Table S13**. Breathing feature analysis –feature mean (e.g. mean respiratory rate) per night, per patient.


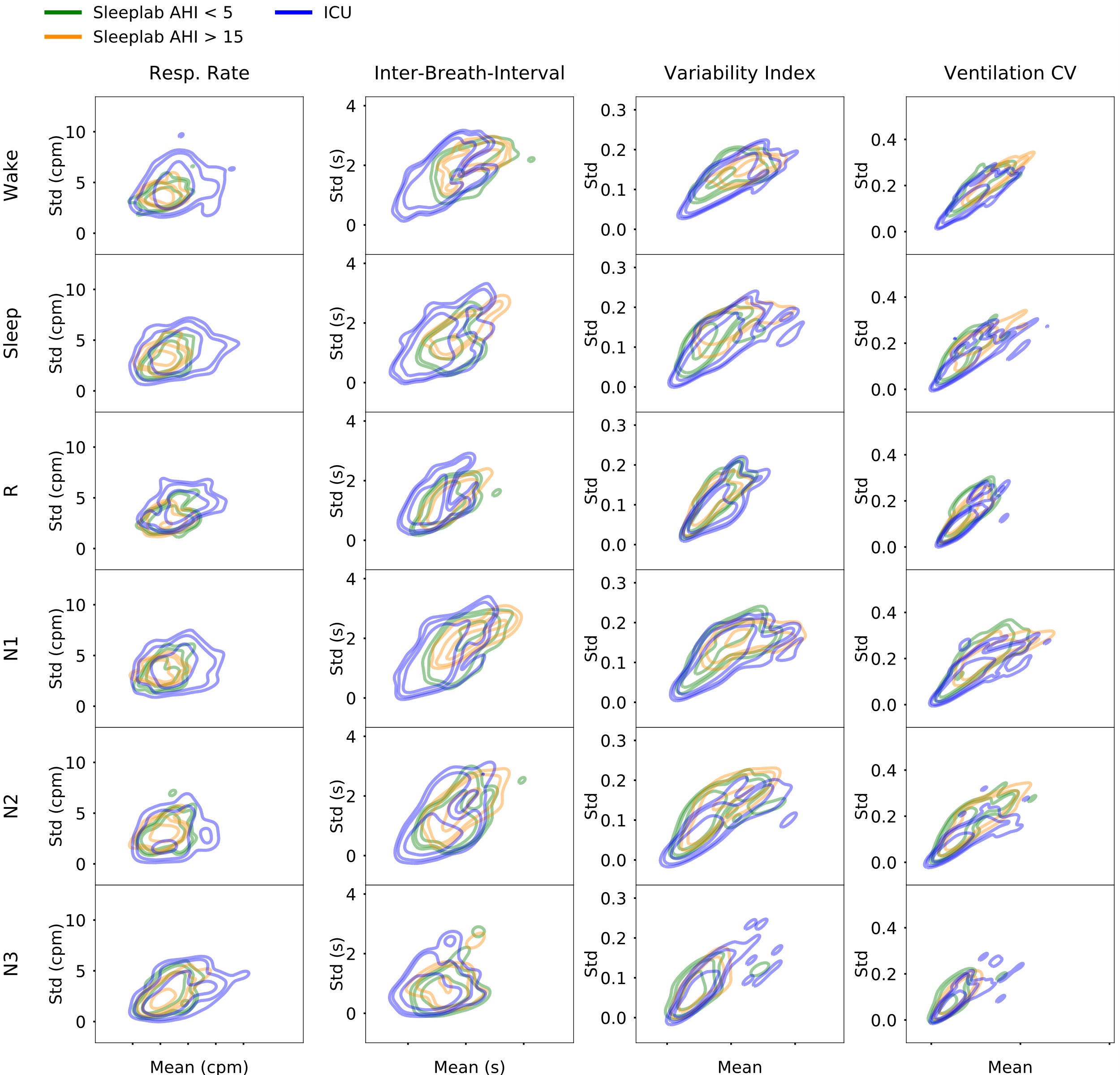


**Figure S6.** Breathing features per night (mean and standard deviation) for each sleep stage and for ICU and sleeplab AHI<5 and AHI>15 cohort. Distributions were fit with a kernel density estimation method, iso mass levels 0.1, 0.2, and 0.5 are shown in the plot.

1. https://clinicaltrials.gov/ct2/show/NCT03355053?term=Investigation+of+Sleep+in+the+Intensive+Care+Unit.

2. Adriana N Vest et al. cliffordlab/PhysioNet-Cardiovascular-Signal-Toolbox: PhysioNet-Cardiovascular-Signal-Toolbox 1.0.2. (Zenodo, 2019). doi:10.5281/zenodo.3401708.

3. McInnes, L., Healy, J. & Melville, J. UMAP: Uniform Manifold Approximation and Projection for Dimension Reduction. arXiv:1802.03426 [cs, stat] (2020).

4. Thomas, R. J., Mietus, J. E., Peng, C.-K. & Goldberger, A. L. An Electrocardiogram-Based Technique to Assess Cardiopulmonary Coupling During Sleep. Sleep **28**, 1151–1161 (2005).

5. Patanwala, A. E., Duby, J., Waters, D. & Erstad, B. L. Opioid conversions in acute care. Ann Pharmacother **41**, 255–266 (2007).

6. MacLaren, R. & Sullivan, P. W. Pharmacoeconomic Modeling of Lorazepam, Midazolam, and Propofol for Continuous Sedation in Critically Ill Patients. Pharmacotherapy: The Journal of Human Pharmacology and Drug Therapy **25**, 1319–1328 (2005).

7. Guina, J. & Merrill, B. Benzodiazepines II: Waking Up on Sedatives: Providing Optimal Care When Inheriting Benzodiazepine Prescriptions in Transfer Patients. J Clin Med **7**, (2018).
